# Generation and analysis of innovative genomically humanized knockin *SOD1*, *TARDBP* (TDP-43), and *FUS* mouse models

**DOI:** 10.1016/j.isci.2021.103463

**Published:** 2021-11-15

**Authors:** Anny Devoy, Georgia Price, Francesca De Giorgio, Rosie Bunton-Stasyshyn, David Thompson, Samanta Gasco, Alasdair Allan, Gemma F. Codner, Remya R. Nair, Charlotte Tibbit, Ross McLeod, Zeinab Ali, Judith Noda, Alessandro Marrero-Gagliardi, José M. Brito-Armas, Chloe Williams, Muhammet M. Öztürk, Michelle Simon, Edward O'Neill, Sam Bryce-Smith, Jackie Harrison, Gemma Atkins, Silvia Corrochano, Michelle Stewart, Jonathan D. Gilthorpe, Lydia Teboul, Abraham Acevedo-Arozena, Elizabeth M.C. Fisher, Thomas J. Cunningham

**Affiliations:** 1 Department of Neuromuscular Diseases, UCL Institute of Neurology, Queen Square, London WC1N 3BG, UK; 2 UK MRC Harwell Institute, Harwell Campus, Oxfordshire OX11 0RD, UK; 3 Research Unit, Hospital Universitario de Canarias; ITB-ULL and CIBERNED, 38320 La Laguna, Spain; 4 Department of Integrative Medical Biology, Umeå University, 901 87, Umeå, Sweden

**Keywords:** Neurogenetics, Neuroscience, Model organism

## Abstract

Amyotrophic lateral sclerosis/frontotemporal dementia (ALS/FTD) is a fatal neurodegenerative disorder, and continued innovation is needed for improved understanding and for developing therapeutics. We have created next-generation *genomically humanized* knockin mouse models, by replacing the mouse genomic region of *Sod1*, *Tardbp* (TDP-43), and *Fus*, with their human orthologs, preserving human protein biochemistry and splicing with exons and introns intact. We establish a new standard of large knockin allele quality control, demonstrating the utility of indirect capture for enrichment of a genomic region of interest followed by Oxford Nanopore sequencing. Extensive analysis shows that homozygous humanized animals only express human protein at endogenous levels. Characterization of humanized FUS animals showed that they are phenotypically normal throughout their lifespan. These humanized strains are vital for preclinical assessment of interventions and serve as templates for the addition of coding or non-coding human ALS/FTD mutations to dissect disease pathomechanisms, in a physiological context.

## Highlights

• Replacement in mice of *Sod1*, *Tardbp*, and *Fus* with orthologous human genomic sequence
• Thorough allele QC of knockin alleles using targeted long-read sequencing
• Mice are viable, fertile, and provide refined tools for ALS/FTD research
• Aging of *hFUS* mice reveals no overt phenotype and minimal transcriptomic disruption

## Graphical abstract

**Figure:**
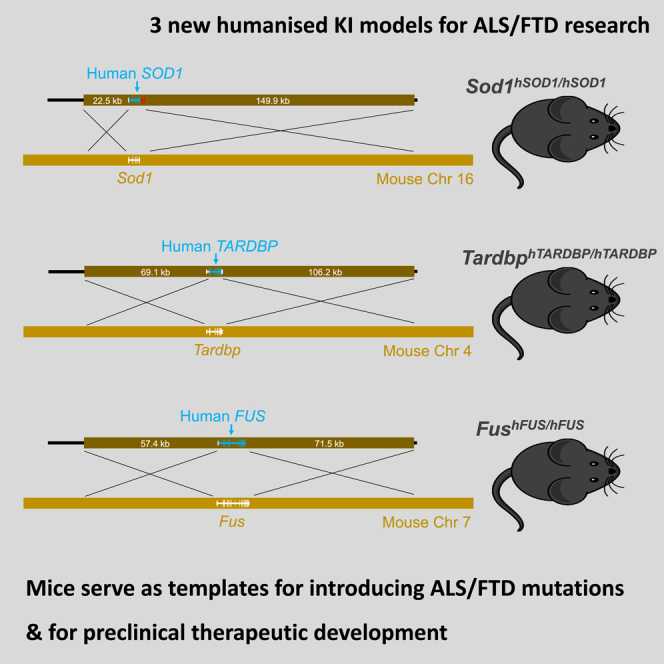

## Introduction

Amyotrophic lateral sclerosis (ALS) is a relentless and devastating neurodegenerative disease that causes the progressive death of motor neurons, resulting in spreading paralysis and death typically within 5 years from diagnosis (Brown and Al-Chalabi, 2017; Hardiman et al., 2017). The lifetime risk for developing ALS is 1 in 300 in the UK (Alonso et al., 2009). ALS and frontotemporal dementia (FTD) lie on a disease spectrum with overlapping genetics, pathology, and symptoms (Abramzon et al., 2020). Most ALS diagnoses occur in mid-life but a wide span of ages has been reported, from adolescence to old age. ALS/FTD remains incurable and essentially untreatable, with two FDA approved ALS treatments that only confer, on average, a few more months of life (Brown and Al-Chalabi, 2017; Hardiman et al., 2017). The majority of ALS is sporadic (sALS), of predominantly unknown cause, but ∼10% is familial (fALS), usually with an autosomal dominant mode of inheritance, with at least 30 possible monogenic forms described in several genes with varying functions.

Mutations in superoxide dismutase 1 (*SOD1*) were the first to be identified in patients with ALS in 1993 (Rosen et al., 1993), but after >25 years of research, we still do not know how mutations in *SOD1* or those identified in other genes lead to selective neuronal death, or what shared or distinct mechanisms are at play between different genetic forms (Mejzini et al., 2019; Taylor et al., 2016). *SOD1* mutations make up ∼20% of fALS—the second most frequent known genetic cause (Brown and Al-Chalabi, 2017). The leading cause for ALS and FTD is a hexanucleotide repeat expansion in intron 1 of *C9orf72* (DeJesus-Hernandez et al., 2011; Renton et al., 2011), while mutations in several RNA-binding proteins including fused in sarcoma (FUS) and TAR DNA-binding protein 43 (TDP-43, encoded by the *TARDBP* gene) indicate that disruption of RNA metabolism is a key pathomechanism (Zhao et al., 2018). *FUS* mutations, occurring in <5% of fALS, may cause an unusually early onset—patients as young as 11 years of age have been described, leading to a particularly aggressive disease (Picher-Martel et al., 2020). *FUS* mutations can also (albeit more rarely) cause FTD, highlighting the continuum between the two diseases (Abramzon et al., 2020). Additionally, FUS pathological aggregates (together with aggregates of related FET-family RNA binding proteins) in the absence of mutations, have been identified in ∼10% of FTD cases (Neumann et al., 2009; Urwin et al., 2010)*.*

While mutations in *TARDBP* are also relatively rare (<5% fALS), the presence of TDP-43 pathology plays a central role in ALS. In ∼97% of all ALS cases, this normal nuclear RNA-binding protein aggregates into ubiquitinated and hyperphosphorylated forms in the cytoplasm of affected cells, leading to an almost complete loss of nuclear function, affecting the normal splicing of hundreds of exons (Hardiman et al., 2017; Suk and Rousseaux, 2020). TDP-43 pathology is described in a growing number of diseases collectively known as TDP-43 proteinopathies, including ALS, ∼45% of FTD cases, and a recently described subtype of old-age dementia called limbic-predominant age-related TDP-43 encephalopathy (LATE) (de Boer et al., 2020; Hardiman et al., 2017; Nelson et al., 2019). A number of key mechanisms have been proposed for how mutations in *TARDBP* or *FUS* cause neurodegeneration from pathological protein aggregation to disrupted RNA metabolism. Interestingly, TDP-43 pathology is not present in SOD1-ALS, suggesting that at least at the pathological level they may be caused by distinct mechanisms (Mackenzie et al., 2007).

The mouse is the mammalian organism of choice for *in vivo* modeling of the complex biology of ALS/FTD, and many mouse models have been invaluable for highlighting effects of mutations (De Giorgio et al., 2019). As fALS is mainly caused by dominant mutations, transgenic mice overexpressing human ALS/FTD genes have been the most widely used models, because they recapitulate human biochemistry of the protein of interest and can model end-stage ALS within a short timeframe. However, in all transgenic strains, the exogenous DNA randomly inserts into the genome and almost always concatemerises, even with large sequences from bacterial artificial chromosome (BAC) donor vectors. Thus, the exogenous DNA disrupts sequences at the insertion site, which may have phenotypic outcomes unrelated to the transgenic sequence: a study of 40 commonly used strains of transgenic mice found at least 75% had altered DNA sequence around the site of insertion, including large deletions (up to a megabase) and structural changes that in half of cases disrupt at least one coding gene (Goodwin et al., 2019). Ectopic expression at non-endogenous loci may also affect spatiotemporal expression patterns and pathogenesis in unpredictable ways unrelated to human disease.

As exogenous DNA usually inserts in multiple copies, gene dosage is altered, which usually affects protein levels derived from the transgene. Sometimes, as in the case of the widely used SOD1 G93A transgenic model of ALS, this can be an advantage and a high protein level mediates the fast onset of phenotypes, enabling researchers to study disease trajectory; within ∼5 months these animals reach the humane endpoint. However, with multiple copies, allele instability can occur within transgene arrays, and stochastic changes in copy number can dramatically alter phenotype (Acevedo-Arozena et al., 2011; Alexander et al., 2004).

Furthermore, phenotypes may be due to overexpression, and not from the effects of mutation. The RNA-binding proteins TDP-43 and FUS are dosage-sensitive; and phenotypes arise from even mild overexpression of the wild-type human gene (Ling et al., 2019; Mitchell et al., 2013; Wils et al., 2010; Xu et al., 2010) and even low copy transgenics have to be bred to mice with the endogenous gene knocked out, in order to keep dosage as normal as possible (Lopez-Erauskin et al., 2018). While crossbreeding to endogenous gene knockout (KO) mice is an added complication for transgenic models, it is necessary to model loss of function, which has recently gained traction as being important for pathomechanism in forms of ALS (Briese et al., 2020; Brown et al., 2021; Humphrey et al., 2020; Klim et al., 2019; Ma et al., 2021; Melamed et al., 2019; Saccon et al., 2013). Finally, transgenic models that ectopically express a wild-type human ALS gene are used as controls for transgenics that express the mutant protein; however, since transgenics integrate at random in different copy numbers, comparisons between control and mutant transgenics are confounded.

Issues of insertional mutagenesis, ectopic overexpression, allele instability, the need to investigate loss of function, gene dosage-sensitivity, and lack of precise genetic control animals, may all now be addressed by the generation of physiologically relevant knockin (KI) strains harbouring mutations in endogenous mouse genes. Such mice express proteins/mutations of interest at endogenous levels, modeling human disease. For ALS genes, this usually leads to mild phenotypes with late disease onset, making them good models to dissect early disease mechanisms (De Giorgio et al., 2019). We and others have made KI lines for the key ALS genes *Sod1* (Joyce et al., 2015), *Tardbp* (Fratta et al., 2018), and *Fus* (Devoy et al., 2017) — all with late onset motor neuron degeneration — that have been used for dissection of ALS disease mechanisms.

However, most KI strains express the mouse protein of interest, thus not modeling human biochemistry that can be important for proteinopathies; for example, human SOD1 protein has distinct biochemical properties associated with specific residues that are not present in the mouse, but are important for SOD1 misfolding, aggregation, and inferring neuronal toxicity in the context of ALS (Crown et al., 2020; DuVal et al., 2019; Nagano et al., 2015; Perri et al., 2020). Thus, our rationale for *genomic humanization* in mice—in which the mouse endogenous gene is precisely replaced with the human orthologous sequence, exons and introns included, and driven by the mouse promoter—is to maintain endogenous expression levels while conferring human protein biochemistry together with human splicing patterns (Nair et al., 2019; Zhu et al., 2019).

Here, we present three genomically humanized KI lines: *Sod1*^*hSOD1/hSOD1*^, *Tardbp*^*hTARDBP/hTARDBP*^, and *Fus*^*hFUS/hFUS*^ (referred to as *hSOD1*, *hTARDBP*, and *hFUS*). Each strain was produced by homologous recombination with large homology arms (22–150 kb) in mouse embryonic stem (ES) cells, allowing us to replace the entire mouse coding sequence with its human equivalent. The generation of these mice is technically challenging and requires extensive quality controls to assess allele integrity and integration at the correct endogenous locus. We present a robust genomic pipeline using indirect capture technology to enrich for high molecular weight (HMW) DNA from the targeted loci followed by long-read sequencing that allows us to validate that our humanization strategy occurred correctly, with precise integration of the targeting vector in the correct endogenous mouse locus. We can also map the recombination events between the vector and the mouse genome. All three human genes functionally replace their mouse orthologs leading to the expression of only human protein at endogenous levels in homozygous humanized mice. Moreover, as a proof of principle, we show extensive phenotypic and molecular characterization of the homozygous *hFUS* mice, showing that they are phenotypically normal throughout their entire lifespan, underscoring the fact that human *FUS* can functionally replace the mouse gene throughout the aging process. These mice will be freely available to the community for driving forward novel findings in ALS/FTD and associated therapeutics.

### Design

#### Humanization strategies

For each of the three genomic humanization KI projects, the principle goal was to achieve endogenous expression of human genes in mouse. In each case, we maintained the endogenous mouse promoter to drive expression of the inserted human genes, with the transition from mouse-to-human sequence beginning at the translational start codon ATG sequence; the rationale being that it was best to maintain coupling of mouse transcriptional machinery with the mouse promoter to drive each human gene at as close to physiological levels as possible. All humanization projects entailed KI of genomic human sequence (i.e. genomic humanization) including all coding exons and intervening introns. Introns were included (rather than a simpler KI of cDNA) to prevent undesired disruption to physiological expression, for example, due to intronic autoregulatory elements (Humphrey et al., 2020). Including *human* introns serves two key purposes: first, it enables modeling of known human intronic variants and mutations, and second, it maintains human splicing complexity and affords study of human gene regulation. For the 3’ end of each locus, we employed a bespoke approach for each gene.

**Figure 1:**
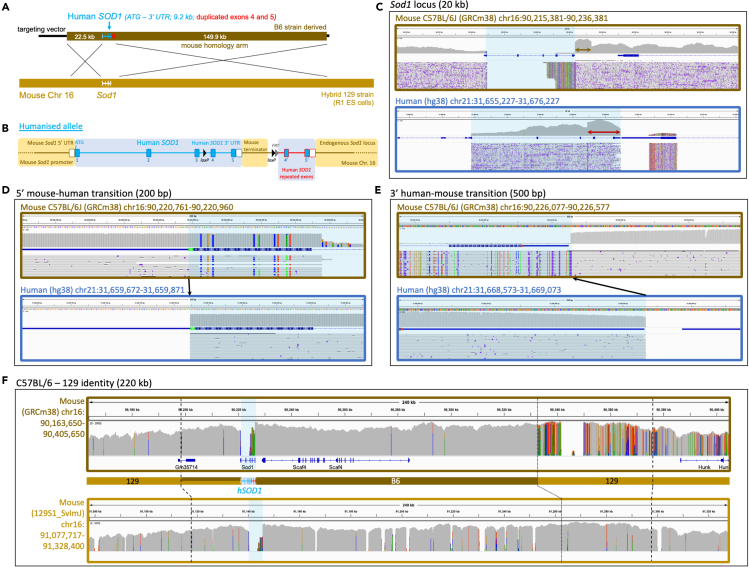
Genomic humanization of the endogenous mouse *Sod1*gene (A) A BAC construct, engineered to harbor the human *SOD1* genomic sequence (blue) flanked by large regions of mouse homology (brown), was used as a donor to replace endogenous mouse *Sod1* with human *SOD1* in mouse ES cells via homologous recombination. (B) The humanized *SOD1* allele in more detail, showing humanization of *Sod1* from the ATG start codon to the end of the 3′ UTR (blue); human exon 4, intron 4, exon 5, the 3 ’UTR, plus 1 kb of downstream mouse terminator sequence was floxed and duplicated to generate a conditional cassette. An *FRT* site downstream of the second *loxP* site is a remnant of selection cassette removal. (C–F) IGV visualizations of Oxford Nanopore read alignments via minimap2. Alignment of nanopore sequencing reads across (C) the mouse and human *SOD1* gene loci, (D) the mouse and human *SOD1* ATG start codon, (E) the mouse and human *SOD1* 3′ UTR, and (F) the wider *SOD1* locus comparing B6 and 129 mouse strain identity, with dashed lines representing the boundaries of the homology arm region and dotted lines delineating B6 and 129 strain genome identity. Blue shading in alignment screenshots represents the humanized region. Brown and red arrows in (C) denote the engineered duplications. Black arrows in (D) and (E) show the precise transition from mouse-to-human and human-to-mouse sequence.

For *hSOD1*, a complex conditional allele was engineered, incorporating a duplication of exon 4, intron 4, exon 5, the human 3’ UTR, and ∼1 kb of downstream mouse terminator sequence (Figures 1A and 1B). In this configuration, the human gene is transcribed and translated as normal, not incorporating the downstream duplicated sequence. The upstream copy of this sequence is flanked by *loxP* sites, such that following CRE recombination, the downstream copy of the sequence (exons 4′ to 5′) is brought into frame and is expressed. This allows for conditional expression of mutations, or reversion from mutation to wild-type, of mutations placed in the downstream or upstream duplicated exons, respectively.

**Figure 2:**
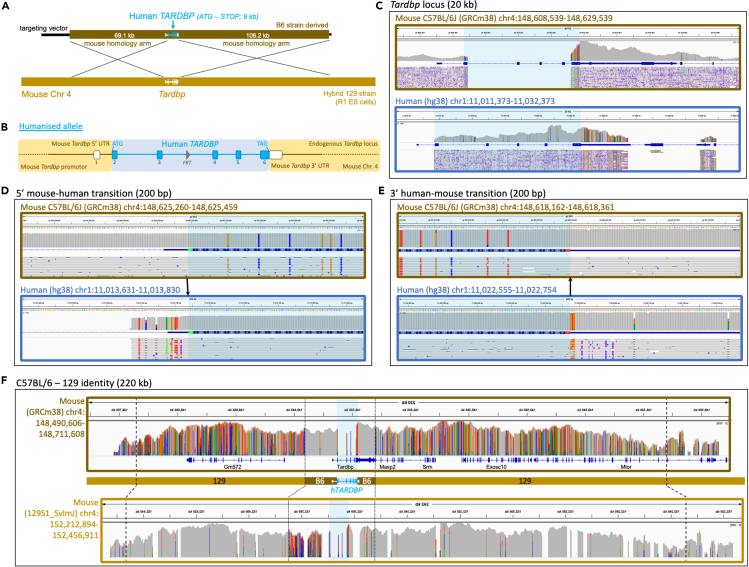
Genomic humanization of the endogenous mouse *Tardbp* gene (A) A BAC construct, engineered to harbor the human *TARDBP* genomic sequence (blue) flanked by large regions of mouse homology (brown), was used as a donor to replace endogenous mouse *Tardbp* with human *TARDBP* in mouse ES cells via homologous recombination. (B) The humanized *TARDBP* allele in more detail, showing humanization of *Tardbp* from the ATG start codon to the TAG stop codon (blue). An *FRT* site in intron 3 is a remnant of selection cassette removal. (C–F) IGV visualizations of Oxford Nanopore read alignments via minimap2. Alignment of nanopore sequencing reads across (C) the mouse and human *TARDBP* gene loci, (D) the mouse and human *TARDBP* ATG start codon, (E) the mouse and human *TARDBP* stop codon, and (F) the wider *TARDBP* locus comparing B6 and 129 mouse strain identity, with dashed lines representing the boundaries of the homology arm region and dotted lines delineating B6 and 129 strain genome identity. Blue shading in alignment screenshots represents the humanized region. Black arrows in (D and E) show the precise transition from mouse-to-human and human-to-mouse sequence.

For *hTARDBP*, a simpler approach was taken and humanization extends only from the start ATG in exon 2 to the stop codon sequence in exon 6 (Figures 2A and 2B). As 3′ UTR disruption has been shown to affect expression of the downstream gene *Masp2* (Dib et al., 2014), which lies tail-to-tail with *Tardbp* so that both overlap in their 3′ UTRs, we chose to keep the endogenous mouse 3′ UTR sequence to maintain correct *Masp2* expression.

**Figure 3:**
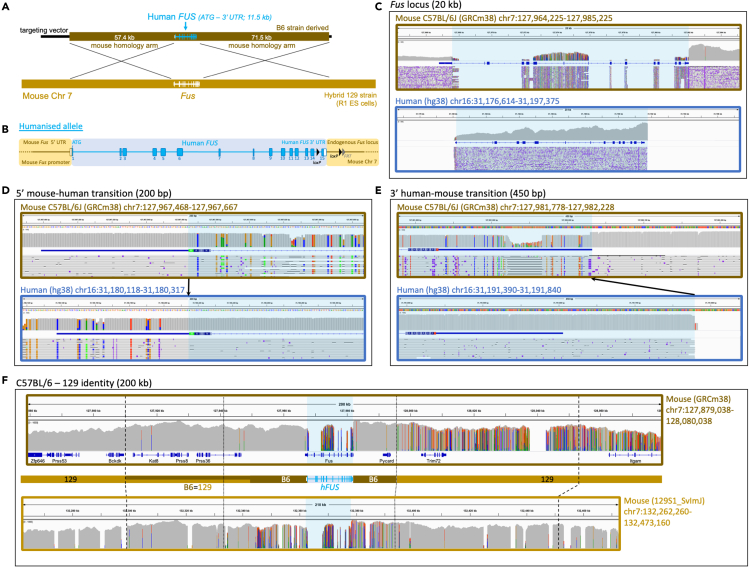
Genomic humanization of the endogenous mouse *Fus* gene (A) A BAC construct, engineered to harbor the human *FUS* genomic sequence (blue) flanked by large regions of mouse homology (brown), was used as a donor to replace endogenous mouse *Fus* with human *FUS* in mouse ES cells via homologous recombination. (B) The humanized *FUS* allele in more detail, showing humanization of *Fus* from the ATG start codon through to the end of the 3′ UTR, including all coding exons and introns, driven by the endogenous *Fus* promoter. A *loxP/FRT* site 1 kb downstream of the 3′ UTR is a remnant of selection cassette removal. A second *loxP* site is also present in the last intron. (C–F) IGV visualizations of Oxford Nanopore read alignments via minimap2. Alignment of sequencing reads across (C) the mouse and human *FUS* gene loci, (D) the mouse and human *FUS* ATG start codon, (E) the mouse and human *FUS* 3′ UTR, and (F) the wider *FUS* locus comparing B6 and 129 mouse strain identity, with dashed lines representing the boundaries of the homology arm region and dotted lines delineating B6 and 129 strain genome identity. Blue shading in alignment screenshots represents the humanized region. Black arrows in (D) and (E) show the precise transition from mouse-to-human and human-to-mouse sequence.

In the case of *hFUS,* the transition from human to mouse was placed after the 3′UTR (Figures 3A and 3B). The human 3′ UTR was included because (1) variants in the 3′ UTR have been linked to risk for developing ALS (Dini Modigliani et al., 2014; Sabatelli et al., 2013) and (2) ALS-frameshift mutations at the 3′ end of FUS incorporate sequence from the human 3′ UTR and if not humanized, such mutations would result in longer 3′neopeptides that may impact pathogenicity (An et al., 2020). In addition, *loxP* sites were placed upstream of exon 15 and downstream of the 3′ UTR to allow for future conditional studies.

#### Validation approach for engineered alleles

Humanized targeting constructs for all three alleles were engineered from large BAC targeting vectors using recombineering technology (Copeland et al., 2001), with the removed mouse sequence ranging from 5.5 kb to 14.5 kb and the KI replacement sequence (after selection cassette removal) ranging from 9 kb to 11.5 kb (Figures 1A, 2A, and 3A). Constructs were electroporated into R1 ES cells, and correctly targeted clones (validated by quantitative PCR copy-count assays, data not shown) were karyotyped and injected into donor blastocysts for mouse production. Considering the relatively large size of each KI and the large homology arms used in gene targeting (together spanning between 140 kb and 184 kb for each allele), standard techniques such as long-range PCR, Sanger sequencing, or Southern blotting were insufficient to validate allele integrity. To be certain of structural and sequence-level allele integrity at the correct locus, we reasoned that high-coverage long-read sequencing data was required. Therefore, we sought to establish the utility of Xdrop (Samplix) indirect target locus enrichment (Blondal et al., 2021) followed by Oxford Nanopore sequencing for large, complex allele validation. As further validation, and to corroborate DNA sequencing data, we analysed mRNA and protein expression in several tissues in all three lines. In addition, for the *hFUS* line, we took validation a step further and performed both transcriptomic and behavioral analyses.

## Results

### Xdrop indirect target locus enrichment and nanopore sequencing to confirm allele integrity in humanized mice

For long-read sequencing allele validation, we first sought to establish mice homozygous for each allele. All three new strains are viable and healthy in homozygosity, with progeny produced with the expected Mendelian ratios (data not shown). Cohorts for all analyses, including homozygotes for sequencing, were produced by intercrossing heterozygous humanized mice, maintained on a C57BL/6J background, with the selection cassette removed for h*SOD1* and h*FUS*, but still present for the more recently established *TARDBP* line.

High molecular weight genomic DNA from homozygous humanized animals was prepared for indirect target locus enrichment using Xdrop™ technology (Samplix) followed by Oxford Nanopore sequencing (Blondal et al., 2021). Briefly, this approach encapsulates high molecular weight DNA into droplets, followed by droplet PCR using detection primers for the loci of interest facilitating fluorescence-activated cell sorting (FACS) sorting of droplets containing the desired loci. Subsequently, droplet-based multiple displacement amplification provides sufficient DNA for nanopore sequencing with high coverage (Figure S1). For each humanization project, we designed between 3 and 4 pairs of detection primer sequences (Table S1) across each respective mouse target loci, used to enrich genomic DNA from the surrounding locus, reaching beyond the extent of the long homology arms (>20 kb) to scrutinize any potential undesired recombination events. This was successfully achieved, with an island of coverage spanning between 200 and 300kb for each locus, with coverage peaking around detection sequences (ranging from 50× to >1,000×) (Figures S2–S4).

Alignment (Minimap2 (Li, 2018)) of reads to the C57BL/6J (GRCm38.p6) reference genome across each targeted gene showed, in each case, a loss of the targeted mouse gene, while alignment to the human (hg38) reference genome showed an island of coverage representing the inserted human sequence (Figure 1, Figure 2, Figure 3). While two of the lines (*hSOD1* and *hTARDBP*) utilized C57BL/6N-derived targeting constructs, we did not align to the C57BL/6N reference owing to lack of differences between C57BL/6J and C57BL/6N at these loci, but also because the C57BL/6J reference is the most complete available, with no gaps. The immediate sequence surrounding the mouse-human and human-mouse boundaries at the 5′ and -3′ ends of the loci has sufficient sequence conservation to align to the opposing species alignment, which revealed the expected divergences (colored lines) from each reference genome, and showed that the intended precise transitions have been incorporated into the genome (Figures 1D, 1E, 2D, 2E, 3D, and 3E). The intended precise insertion can also be demonstrated by alignment of reads to a sequence file of the expected engineered allele (Figures S5–S7). This revealed uninterrupted alignment profiles, in contrast to mouse and human reference alignments, with engineered insertions including *loxP* and *FRT* sites clearly visible in the alignment tracks (Figures S5–S7).

Large engineered structural variants in the *hSOD1* allele—duplication of the 3′ human sequence, plus duplication of the mouse terminator sequence— were clearly visible as sharp 2-fold steps in coverage in the profiles of human and mouse alignments; while alignment to the expected allele resolved these coverage anomalies (Figures 1C and S5).

Two anomalies in the alignment profile against the human reference genome, in introns 2 and 5 of the *hTARDBP* allele, respectively, warranted further investigation (Figure S6; red arrows, region X and Y). Each region exhibited a concentration of base positions with high error rates, although at each position, the correct base was called in the majority (>70% for each affected nucleotide). Additionally, region Y in intron 5 exhibited an anomalous step-up in coverage. Both regions map precisely to the boundaries of highly repetitive SINE elements (Figure S8); we suspected mapping error as the majority of reads mapped correctly, many of which mapped beyond the boundaries of these features on both sides. Alignment with alternative software, NGMLR (Sedlazeck et al., 2018), built specifically to correctly identify structural variants (versus minimap2, built for fast alignment), resolved the alignment anomalies (Figure S6). We also PCR amplified and Sanger sequenced intron 5 using various primer sets, observed the expected band sizes, and found no evidence of sequence anomalies corresponding to the nucleotide positions with high error rates via nanopore sequencing, or in any other position (data not shown).

Alignment of reads to the C57BL/6J reference genome across the wider targeted locus for each mouse line showed that while the human insertion and proximal-most regions align as expected, more distal homology arm correspondent regions have evidence of misalignment (Figures 1F, 2F, 3F; top panels). Our strategy used 129 strain ES cells with targeting vector homology arms derived from the C57BL/6J or C57BL/6N (herein referred to as B6) genetic background, followed by backcrossing all to C57BL/6J, and so we additionally aligned reads from each mouse to the available 129S1_SvImJ reference genome for comparison (Figures 1F, 2F, 3F; bottom panels). This demonstrated the transitions from B6 identity to 129 strain identity (where SNPs polymorphic for both strains exist) and thus pinpointed the homologous recombination breakpoints (i.e. the extent of flanking sequence from the targeting vector that recombined into the respective loci together with the humanized genes). This is most clearly demonstrated in the *hTARDBP* allele, where B6 and 129 strains are divergent on both homology arms (Figure 3F). 11 kb (5′ to *hTARDBP*) and 5.5 kb (3′ to *hTARDBP*) of the proximal homology arm correspondent regions are derived from the B6 targeting vector, while the more distal homology arm correspondent regions are clearly 129-derived, showing that only a minor fraction (16.5/175 kb total) of the homology arms recombined into the locus together with the *hTARDBP* gene (Figures S9 and S10).

For the *hFUS* allele, ∼30 kb of the proximal 5′ homology arm correspondent region is clearly derived from the B6 targeting vector, while the remaining distal 27 kb of the 5′ homology arm has no appreciable divergence between strains to make a clear determination (Figure S11). For the *hFUS* 3′ homology arm region, the proximal 15 kb is B6-derived with the exception of a single SNP that maps to the 129 strain, suggesting that recombination did not occur cleanly at a single point (Figure S12). The distal 56.5 kb of the *hFUS* 3′ homology arm region is clearly 129 strain-derived. For *hSOD1*, the relatively shorter 22.5 kb 5′ homology arm region is conserved between B6 and 129 strains, while in the 3′ homology arm region where divergent sequence exists, albeit sparsely, the homologous recombination breakpoint is ∼100 kb from *hSOD1* with the distal 50 kb clearly mapping to 129 strain (Figure S13).

In all three humanization projects, we observe seamless alignment stretching beyond the limit of the homology arm regions and together with the clear 129-B6 transitions that show recombination breakpoints, we can be confident that the desired integrations occurred at the correct loci.

After accounting for the humanization event, engineered insertions, the anomalies in *hTARDBP* introns 2 and 5, and strain differences, very few minor alignment imperfections remain. A number of gaps (of varying sizes) lie within the 129 strain alignments, including where the sequence is evidently of 129 strain identity. These all map to gaps in the 129 Reference Genome (sequence denoted by strings of Ns) rather than any untoward misalignment. In addition, there a number of known (human population) SNPs present in the human gene sequence in the *hSOD1* and *hTARDBP* targeting vectors, all within non-coding regions, which differ from the respective reference human and mouse genomes as reflected in the alignments (marked by #; Figures S5 and S6). Finally, we saw positions in which individual nucleotides were flagged (colored lines) because they had an increased error rate in the sequencing with respect to the reference sequence. Such positions are all in non-coding regions, are highly polymorphic, and map to homopolymer sequences, low complexity sequences, and repetitive element regions, which are a known cause for higher error rates in long-read sequencing data.

### Humanized mouse strains express human, not mouse, gene products

**Figure 4:**
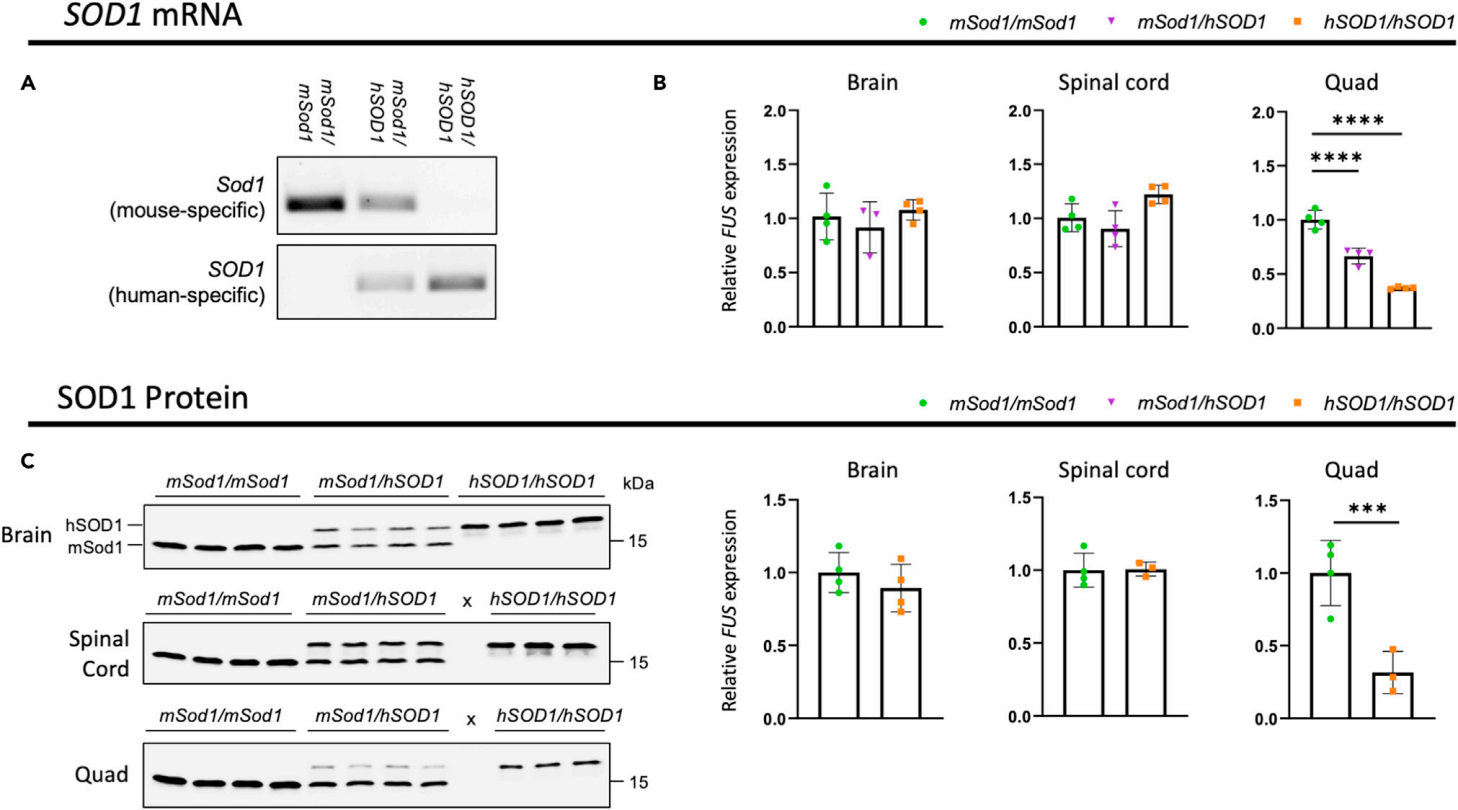
*hSOD1/hSOD1* mice only express human *SOD1* (A) RT-PCR in brain from 4-month-old male mice using mouse-specific *Sod1* and human-specific *SOD1* primers. (B) Quantitative RT-PCR using conserved mouse-human *Sod1*/*SOD1* primers in tissues from male 4-month-old mice (n = 3–4 per genotype). Mean ± SD, One-way ANOVA with Dunnett's post hoc test. (C) Immunoblots using a pan-mouse-human SOD1 antibody in tissues from male 4-month-old mice (n = 3–4 per genotype). Human SOD1 has lower mobility than mouse Sod1 in SDS-PAGE and appears as a higher band. Heterozygote quantification not included for protein data owing to confounding issue of double bands. Blots normalized to total protein (Figure S18). Unused lanes marked by x. Mean ± SD, Unpaired t test. ∗p< 0.05, ∗∗∗p< 0.001, ∗∗∗∗p< 0.0001.

**Figure 5:**
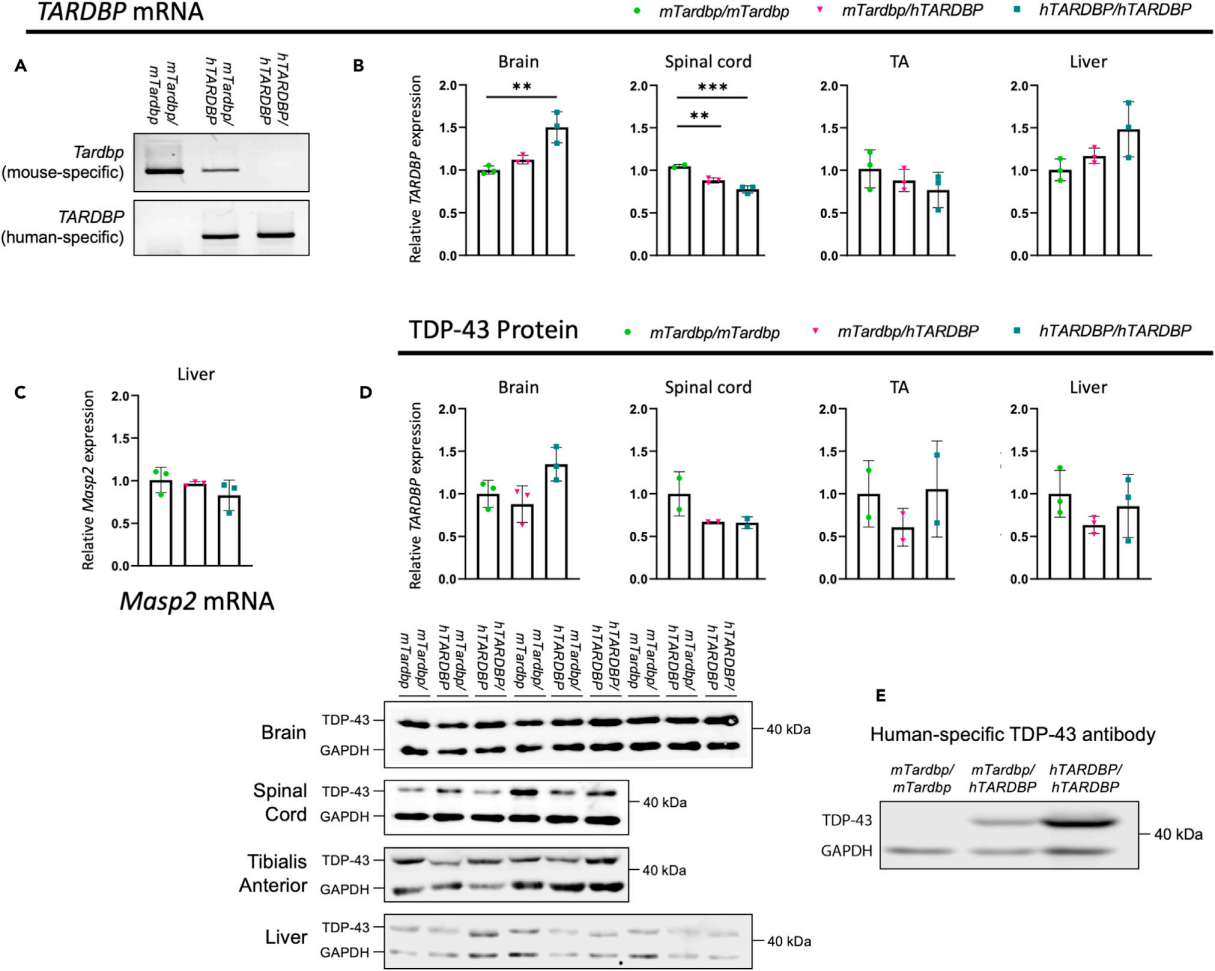
*hTARDBP/hTARDBP* mice only express human *TARDBP* All tissues analyzed are from female 14-week-old *hTARDBP* mice and controls. (A) RT-PCR in brain using mouse-specific *Tardbp* and human-specific *TARDBP* primers. (B) Quantitative RT-PCR using conserved mouse-human *Tardbp*/*TARDBP* primers (n = 2–3 per genotype per tissue). (C) Quantitative RT-PCR using mouse *Masp2* primers in liver (n = 3 per genotype). (D) Immunoblots using pan-mouse-human TDP-43 antibody (n = 2–3 per genotype). (E) Immunoblot using human-specific TDP-43 antibody in brain. ∗∗p< 0.01, ∗∗∗p< 0.001, Mean ± SD, One-way ANOVA with Dunnett's post hoc test.

**Figure 6:**
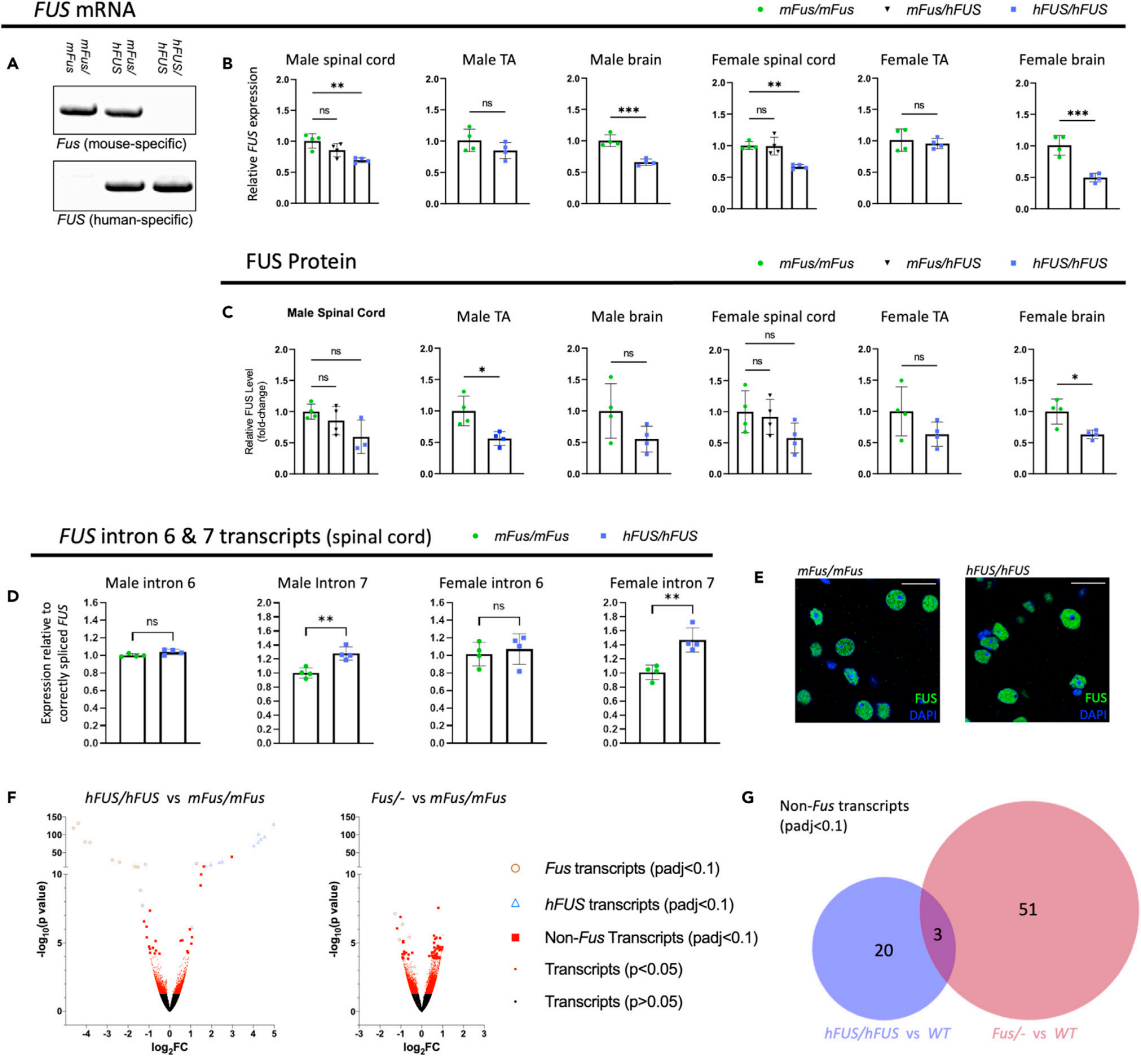
*hFUS/hFUS* mice only express human *FUS* but have minimal transcriptomic disruption (A) RT-PCR from E14.5 embryonic brain using mouse-specific *Fus* and human-specific *FUS* primers. (B) Quantitative RT-PCR using conserved mouse-human *Fus*/*FUS* primers in tissues from male and female 18-month-old mice (n = 3–4 per genotype per sex). (C) Quantification of immunoblots using pan-mouse-human FUS antibody in male and female spinal cord, brain, and tibialis anterior (TA) muscle tissue collected from mice aged 18 months (n = 4 per genotype per sex). Blots normalized to GAPDH (Figure S18). (D) Quantitative RT-PCR using conserved mouse-human *Fus/FUS* primers to determine levels of intron 6 and intron 7 containing FUS transcripts, relative to correctly spliced *FUS* in male and female 18-month-old spinal cord (n = 4 per genotype per sex). Data presented as mean ± SD, ns = not statistically significant, ∗ = p ≤ 0.05, ∗∗ = p ≤ 0.01, ∗∗∗ = p ≤ 0.001, calculated using One-way ANOVA with Dunnett's post hoc test or Student's T test. Heterozygotes only included for spinal cord expression data. (E) Immunofluorescent staining of FUS (green) in 8-month-old motor cortex showing nuclear localization of FUS. Scale bar = 20 μm. (F) RNAseq volcano plots showing the statistical distribution of differentially expressed transcripts between *hFUS*/*hFUS* and *mFus/mFus* (left) and *mFus/-* and *mFus/mFus* (right), from 3-month-old spinal cord tissue (n = 4 per genotype). Significant (padj<0.1) mouse *Fus* transcripts are shown by brown open circles, significant human *FUS* transcripts are shown by blue open triangles, significant non-*Fus* transcripts are shown by red squares, and all other transcripts are shown by red dots (p < 0.05) and black dots (p > 0.05). (G) Venn diagram comparing the number of significant non-*FUS* differentially expressed transcripts between *hFUS/hFUS* and *WT* (blue; left) and *mFus*/- and *mFus/mFus* (red; right). See Data S1 for more RNAseq details.

To complement the long-read sequencing data, we next sought to confirm the expression of human and not mouse gene products in the three lines. Starting with RT-PCR, we designed primers specific to each of the mouse cDNA sequences and primers specific to the human cDNA sequences to specifically test for the loss of mouse and gain of human mRNA in each respective line. In all three lines, we observed loss of mouse mRNA in homozygous humanized animals, absence of human mRNA in wild-type littermates, and gain of human mRNA in heterozygous and homozygous humanized animals (Figures 4A, 5A, and 6A).

Quantitative RT-PCR (qRT-PCR) and complementary immunoblots were then performed from multiple tissue types to quantify expression of the human gene products. Starting with *hSOD1* animals, mRNA and protein levels in the central nervous system (CNS; brain and spinal cord) were not significantly different at 4 months of age (Figures 4B and 4C), suggesting that the insertion of the conditional allele does not affect expression *per se*. In contrast, expression in muscle was significantly reduced at both the mRNA and protein level compared to controls (Figures 4B and 4C), dipping below 50% of wild-type levels, suggesting an interesting muscle-specific alteration in gene regulation that warrants future investigation. Mouse SOD1 protein has evident reduced mobility versus human SOD1, so we were able to further corroborate loss of mouse and gain of human SOD1 expression in homozygous humanized mice (Figure 4C). In SOD1 immunostaining experiments, we observed expected wide distribution of SOD1 localization in both the nucleus and cytoplasm in wild-type and *hSOD1* spinal cords (Figure S14).

In *hTARDBP* animals, at 14 weeks of age, we see minor differences in *Tardbp/TARDBP* mRNA expression levels between homozygous humanized mice and controls in brain and spinal cord, but not in skeletal muscle or liver (Figure 5B). These are not reflected in any significant changes at the TDP-43 protein level in any of the four tissues analyzed (Figure 5D). To further assess TDP-43 functionality, we assessed the splicing of two cassette exons known to be controlled by TDP-43 function (exon 18 of *Sortilin* and exon 5 of *Eif4h*(Fratta et al., 2018)) by RT-PCR from brain and spinal cord and found no differences in exon inclusion between any of the genotypes (Figure S15), suggesting that human TDP-43 protein can functionally replace the mouse protein splicing function. Consistent with this, in TDP-43 immunostaining experiments, we observed expected nuclear distribution of TDP-43 localization in wild-type and *hTARDBP* brain (Figure S15). To verify that human TDP-43 protein is indeed expressed in *hTARDBP* mice, we took advantage of a monoclonal antibody that recognizes human but not mouse TDP-43. As expected, the human-specific antibody only recognized TDP-43 in homozygous and, to a lesser extent, in heterozygous humanized mice (Figure 5E). As mouse *Tardbp* and *Masp2* genes are located tail-to-tail and share their 3′UTR, we assessed *Masp2* expression in liver, where it is mainly expressed, and found that, as expected, its expression was not affected by the humanization strategy leaving the mouse 3′UTR intact (Figure 5C).

For *hFUS* animals, we were able to characterize expression of the humanized gene more extensively in males and females during aging. At 18 months of age, we observed non-significant to mild (statistically significant) reductions in mRNA and protein expression in *hFUS* spinal cord, brain, and TA muscle versus wild-type (Figures 6B and 6C); a finding mirrored at an earlier 3-month timepoint (Figures S16A and S16B). FUS is known to autoregulate via binding to *FUS* introns 6 and 7, leading to intron retention that translates into a non-functional FUS gene product (Humphrey et al., 2020). We therefore designed qRT-PCR splicing assays to quantify levels of intron 6 and 7 retaining transcripts, normalized to correctly spliced *FUS* (exon 7–10), using primer sequences conserved between mouse and human. In both male and female 18-month-old spinal cord samples, we detected significantly increased levels of intron 7 retention, but no difference in intron 6 retention (Figure 6D); this is consistent with mild but significant reduction in *FUS* mRNA expression in hFUS spinal cords at this timepoint, and may reflect an increased propensity for human FUS protein to bind to human *FUS* intron 7, versus mouse. We saw no significant difference in intron 6 or 7 intron retention at 3 months (Figure S16C), consistent with no significant differences between genotypes at this timepoint. To assess for potential changes in hFUS protein levels during the aging process, we ran protein samples from *hFUS* and wild-type male spinal cord at early and late timepoints, together on the same blot. While we saw no change in wild-type FUS over time, consistent with a previous report (Huang et al., 2010), hFUS protein levels showed a significant drop with age (Figure S17). This is consistent with the observed difference in autoregulation and mRNA expression only at the late timepoint in spinal cord, suggesting a possible difference in response to aging between mouse and human *FUS* transcripts.

Mouse FUS protein has fewer amino acid residues versus human FUS (518 versus 526); this is reflected in the immunoblots where band sizes in humanized sample tissue run marginally more slowly than wild-type, providing additional corroboration that human FUS has replaced mouse FUS (Figure S18). In addition, we were able to show that hFUS protein correctly localizes to the nucleus (Figure 6E), in contrast to localization in FUS-ALS and FUS-FTD patients, where FUS protein is found to aggregate in the cytoplasm (Kwiatkowski et al., 2009; Urwin et al., 2010; Vance et al., 2009). In aged, 18-month animals, we observed equivalent and diffuse cytoplasmic FUS staining, together with prominent nuclear staining, in both wild-type and *hFUS* spinal cords, consistent with previous reports of age-related FUS-mislocalization (Figure S16D) (Huang et al., 2010).

Reduction in mRNA is most prominent in *hSOD1* skeletal muscle, and to a lesser extent in *hFUS* muscle. We therefore sectioned muscles from these animals to assess for potential muscle phenotypes (Figure S19). We observed no difference in fiber size or proportion of centrally located nuclei (a marker of muscle regeneration), even in 18-month old homozygous *hFUS* skeletal muscle, suggesting that the observed expression drops in *hSOD1* and *hFUS* mice has limited impact on muscle.

Taken together, while we do observe alterations in expression that may be driven by changes in transcriptional regulation, levels are maintained within a physiological range, especially within the CNS.

### Homozygous *hFUS* mice display minimal transcriptomic disruption

Since FUS is an RNA-binding protein and regulates RNA metabolism on a genome wide scale, we sought to gauge the transcriptomic impact of humanizing mouse *Fus*. We conducted RNAseq of spinal cord tissue at 3 months of age, comparing *hFUS* homozygous mice with wild-type littermates. We included heterozygous KO (*mFus*/-) animals as a measure of mild loss of function. We included human *FUS* transcripts together with the mouse reference assembly when mapping reads; as expected, we saw expression of *hFUS* transcripts and concomitant loss of mouse *Fus* transcripts in *hFUS* animals (Figure 6F, blue triangles and brown circles; Table 1). Taking aside *FUS*/*Fus*-related transcripts, very few transcripts were differentially expressed in *hFUS* animals and fewer in comparison to *mFus/-* spinal cord (Figures 6F and 6G; Tables 1 and 2). Among the 23 non-*Fus* related differentially expressed transcripts (including 2 *Fus* pseudogenes with strong downregulation that we presume to be misaligned with *Fus*) found to be statistically significantly differentially expressed in *hFUS* homozygous spinal cord tissue, 13 are on chromosome 7. These 13 transcripts are sparsely spread across a 30.9 Mb chromosomal region surrounding the *Fus* locus, but not including genes in the immediate vicinity of the *Fus* locus (the closest affected genes are *Gdpd3* and *Tial1*, 1.2 Mb and 472 kb away, respectively). Considering that 129 strain ES cells were used to generate these mice, followed by backcrossing to the C57BL/6J genetic background, we expected a large chromosomal region surrounding the *Fus* locus to remain 129 strain identity through genetic linkage to the targeted locus for which we are selectively breeding. Indeed, whole genome SNP analysis of *hFUS* mice (of the same generation as RNAseq animals) revealed that 46 SNPs of 129 identity (specifically the 129X1/SvJ strain) clustered within a 12.3 Mb span (chr7: 117044155-129357839) of the locus surrounding *hFUS*, encompassing 10 of the 13 differentially expressed non-*Fus* transcripts on chromosome 7 (Data S1). This SNP data strongly suggests that differentially expressed transcripts on chromosome 7 are primarily due to sequence differences between 129 and B6 strains providing even further corroboration that the targeted locus lies in the expected region on chromosome 7. Thus, only 13 differentially expressed transcripts (*Rps15a-ps8-201, Rps15a-ps4-201, Hk1-220, Opa1-203, Arid1b-201, Acot7-206, Nup214-209, Atp2b2-205, Fyttd1-213, Rps13-206, Mgst1-207, Pak1-204,* and *Trim34a-201*) remain after accounting for *Fus*/*FUS* transcripts (that we expected to change) and excluding Fus-locus genetically linked transcripts, reducing the direct overall transcriptomic impact of *Fus* humanization*.* Three transcripts from this list (Hk1-220, Arid1b-201, and Fyttd1-213) were differentially expressed in both *hFUS* homozygous and *mFus/-* spinal cord, versus wild-type, with expression of all three transcripts changing in the same direction, suggesting a mild loss of function effect, at least for these three transcripts, in *hFUS* homozygotes.

**Table 1:** List of differentially expressed transcripts in *hFUS/hFUS* versus *mFus/mFUS* mice

Transcript	Transcript name	Transcript type	Log_2_FC	p value	padj
**Human *FUS* transcripts**					

ENST00000474990	FUS-203	processed_transcript	4.97	1.78E-130	6.71E-126
ENST00000487509	FUS-206	retained_intron	4.24	1.84E-102	3.48E-98
ENST00000380244	FUS-202	protein_coding	4.54	1.38E-94	2.09E-90
ENST00000569760	FUS-212	retained_intron	4.36	1.18E-88	1.49E-84
ENST00000254108	FUS-201	protein_coding	4.23	5.28E-80	4.98E-76
ENST00000568685	FUS-210	protein_coding	4.02	3.80E-70	2.87E-66
ENST00000566605	FUS-209	nonsense_mediated_decay	2.50	5.41E-26	3.14E-22
ENST00000568901	FUS-211	retained_intron	2.37	2.24E-23	1.12E-19
ENST00000483853	FUS-204	retained_intron	1.96	3.83E-17	1.61E-13
ENST00000487045	FUS-205	retained_intron	1.71	3.32E-13	1.32E-09
ENST00000564766	FUS-208	retained_intron	1.06	7.36E-07	0.00185

***Fus* transcripts**					

ENSMUST00000150411	Gm10167-201	processed_pseudogene (Fus)	–4.39	1.12E-132	8.48E-128
ENSMUST00000077609	Fus-201	protein_coding	–4.62	2.87E-119	7.22E-115
ENSMUST00000121616	Fus-204	protein_coding	–4.06	1.22E-80	1.32E-76
ENSMUST00000155941	Fus-211	retained_intron	–3.82	2.37E-79	1.99E-75
ENSMUST00000136289	Fus-207	retained_intron	–2.75	1.09E-30	6.88E-27
ENSMUST00000128851	Fus-206	retained_intron	–2.41	4.70E-24	2.53E-20
ENSMUST00000137464	Fus-208	processed_transcript	1.28	2.57E-20	1.21E-16
ENSMUST00000106251	Fus-203	protein_coding	–1.17	3.65E-18	1.62E-14
ENSMUST00000206627	Gm8309-201	processed_pseudogene (Fus)	–1.68	2.23E-12	8.41E-09
ENSMUST00000174196	Fus-213	retained_intron	–1.53	8.04E-11	2.76E-07
ENSMUST00000205351	Fus-216	retained_intron	–1.40	1.45E-09	4.37E-06
ENSMUST00000205261	Fus-215	retained_intron	–1.31	1.85E-08	5.38E-05

**Non-*Fus* transcripts**					

ENSMUST00000032944b	Gdpd3-201	protein_coding	2.97	2.20E-39	1.51E-35
ENSMUST00000205468^b^	Gdpd3-202	retained_intron	1.63	3.03E-12	1.09E-08
ENSMUST00000206350^b^	Gdpd3-204	retained_intron	1.50	1.02E-10	3.35E-07
ENSMUST00000117914	Rps15a-ps8-201	processed_pseudogene	1.48	6.51E-10	2.05E-06
ENSMUST00000119415	Rps15a-ps4-201	transcribed_processed_pseudogene	–0.95	4.48E-08	0.00013
ENSMUST00000161160	Hk1-220	retained_intron	–1.24	2.69E-07	0.00073
ENSMUST00000172457^b^	Rps15a-207	protein_coding	–1.11	6.54E-07	0.00170
ENSMUST00000084586^b^	Slx1b-201	retained_intron	0.96	1.03E-06	0.00250
ENSMUST00000207147^b^	B230311B06Rik-201	lincRNA	–1.10	3.96E-06	0.00906
ENSMUST00000160101	Opa1-203	retained_intron	1.06	3.86E-06	0.00906
ENSMUST00000092723	Arid1b-201	protein_coding	0.60	6.45E-06	0.01432
ENSMUST00000042942^b^	Sec23ip	protein_coding	0.52	6.98E-06	0.01504
ENSMUST00000167926	Acot7-206	protein_coding	–0.68	7.39E-06	0.01549
ENSMUST00000146138	Nup214-209	retained_intron	1.03	1.13E-05	0.02307
ENSMUST00000144507	Atp2b2-205	processed_transcript	–0.99	1.88E-05	0.03729
ENSMUST00000232272	Fyttd1-213	protein_coding	–0.81	2.30E-05	0.04450
ENSMUST00000206712^a^	Rps13-206	retained_intron	–0.95	2.73E-05	0.05143
ENSMUST00000140932	Mgst1-207	protein_coding	–0.66	3.64E-05	0.06696
ENSMUST00000206351^a^	Pak1-204	protein_coding	0.84	4.14E-05	0.07431
ENSMUST00000060315^a^	Trim34a-201	protein_coding	–0.94	5.31E-05	0.09109
ENSMUST00000128482^b^	Rps15a-203	protein_coding	0.87	5.23E-05	0.09109
ENSMUST00000106588^b^	Rps15a-201	protein_coding	–0.72	5.85E-05	0.09816
ENSMUST00000106226^b^	Tial1-202	protein_coding	–0.50	6.01E-05	0.09863

a Indicates non-*Fus* transcripts on chromosome 7.

b Indicates non-*Fus* transcripts in a region of preserved 129 identity surrounding the *Fus* locus.

**Table 2:** List of differentially expressed transcripts in *mFus/-* versus *mFus/mFUS* mice

Transcript	Transcript name	Transcript type	Log_2_FC	p value	padj
***Fus* transcripts**					

ENSMUST00000136289	Fus-207	retained_intron	–1.28	7.34E-08	0.00151
ENSMUST00000121616	Fus-204	protein_coding	–0.92	4.40E-07	0.00453
ENSMUST00000150411	Gm10167-201	processed_pseudogene (Fus)	–0.59	3.78E-06	0.02042
ENSMUST00000128851	Fus-206	retained_intron	–1.07	5.91E-06	0.02213

**Non-*Fus* transcripts**					

ENSMUST00000077735	Evl-202	protein_coding	0.79	2.81E-08	0.00116
ENSMUST00000232272	Fyttd1-213	protein_coding	–1.02	1.29E-07	0.00178
ENSMUST00000161160	Hk1-220	retained_intron	–1.18	8.82E-07	0.00727
ENSMUST00000072178	Acsl6-203	protein_coding	0.70	4.21E-06	0.02042
ENSMUST00000024042	Creld2-201	protein_coding	0.59	2.98E-06	0.02042
ENSMUST00000105835	Rap1gap-203	protein_coding	0.88	4.46E-06	0.02042
ENSMUST00000180235	Bsg-206	retained_intron	0.64	5.11E-06	0.02106
ENSMUST00000160930	Selenop-207	protein_coding	–0.89	7.57E-06	0.02600
ENSMUST00000183801	Zfp280d-208	nonsense_mediated_decay	–0.90	9.04E-06	0.02659
ENSMUST00000114066	Cpeb2-202	protein_coding	–0.88	8.75E-06	0.02659
ENSMUST00000092723	Arid1b-201	protein_coding	0.59	9.90E-06	0.02719
ENSMUST00000006478	Tmem147-201	protein_coding	0.77	1.62E-05	0.04169
ENSMUST00000172753	Hspa1b-201	protein_coding	0.99	2.02E-05	0.04254
ENSMUST00000176515	Chtf8-203	protein_coding	0.79	2.09E-05	0.04254
ENSMUST00000227478	Hsf1-205	protein_coding	0.82	2.17E-05	0.04254
ENSMUST00000074575	Snrnp70-201	protein_coding	0.60	2.16E-05	0.04254
ENSMUST00000018311	Stard3-201	protein_coding	0.86	1.95E-05	0.04254
ENSMUST00000234180	Mtch1-209	Nonsense-mediated_decay	0.90	2.68E-05	0.04805
ENSMUST00000155957	Ddr1-211	Nonsense-mediated_decay	0.72	2.61E-05	0.04805
ENSMUST00000223361	Cyth2-209	protein_coding	0.95	3.05E-05	0.05245
ENSMUST00000230968	Puf60-211	retained_intron	0.85	3.38E-05	0.05569
ENSMUST00000194596	Bcan-206	retained_intron	0.63	3.60E-05	0.05707
ENSMUST00000230641	Cd47-208	protein_coding	–0.85	4.31E-05	0.06577
ENSMUST00000161567	Pam	protein_coding	–0.45	5.26E-05	0.06778
ENSMUST00000144531	Naa38-204	protein_coding	0.49	5.08E-05	0.06778
ENSMUST00000108899	Acsl6-208	protein_coding	–0.75	5.24E-05	0.06778
ENSMUST00000131286	Ndufs2	processed_transcript	0.50	5.02E-05	0.06778
ENSMUST00000231956	Septin5	protein_coding	0.57	4.88E-05	0.06778
ENSMUST00000124478	Drap1-204	retained_intron	0.72	5.95E-05	0.07429
ENSMUST00000123531	Myo5a-202	Nonsense-mediated_decay	–0.87	6.59E-05	0.07437
ENSMUST00000126586	Kmt2e-203	Nonsense-mediated_decay	–0.84	6.50E-05	0.07437
ENSMUST00000141536	Gpd2-204	processed_transcript	–0.92	6.68E-05	0.07437
ENSMUST00000159283	Manf-202	protein_coding	0.71	6.18E-05	0.07437
ENSMUST00000169694	Pla2g7-206	protein_coding	–0.63	7.22E-05	0.07827
ENSMUST00000063084	Xbp1	protein_coding	0.62	7.64E-05	0.08070
ENSMUST00000160902	Hyou1-204	protein_coding	0.49	8.34E-05	0.08588
ENSMUST00000160978	Manf-205	retained_intron	0.71	8.92E-05	0.08853
ENSMUST00000215296	Cdc37	protein_coding	0.36	9.15E-05	0.08853
ENSMUST00000164479	Stard10-203	protein_coding	0.59	9.24E-05	0.08853
ENSMUST00000225761	Atxn2-220	protein_coding	0.69	9.99E-05	0.09357
ENSMUST00000171044	Mark2-218	retained_intron	0.87	0.00011	0.09366
ENSMUST00000143635	Rragc-203	processed_transcript	–0.92	0.00011	0.09366
ENSMUST00000038614	Ypel3	protein_coding	0.75	0.00011	0.09366
ENSMUST00000113461	Nrxn2	protein_coding	0.55	0.00010	0.09366
ENSMUST00000152288	Emd-206	Nonsense-mediated_decay	0.73	0.00011	0.09366
ENSMUST00000001081	Rmnd5b-201	protein_coding	0.53	0.00012	0.09507
ENSMUST00000037551	Ppp1r16a-201	protein_coding	0.61	0.00012	0.09531
ENSMUST00000232025	Arvcf-212	protein_coding	0.75	0.00013	0.09585
ENSMUST00000124745	Dda1-202	protein_coding	0.67	0.00013	0.09585
ENSMUST00000080936	Med15-202	protein_coding	0.60	0.00013	0.09585
ENSMUST00000153847	Hspa8-209	processed_transcript	0.86	0.00013	0.09585
ENSMUST00000193119	Zfp639-204	protein_coding	–0.89	0.00013	0.09588
ENSMUST00000154954	Mkln1-211	processed_transcript	–0.90	0.00013	0.09588
ENSMUST00000109601	Rab1a-202	protein_coding	–0.66	0.00014	0.09992

### Homozygous *hFUS* mice display no motor or other overt phenotypes throughout aging, and maintain normal motor neuron counts in the lumbar spinal cord

**Figure 7:**
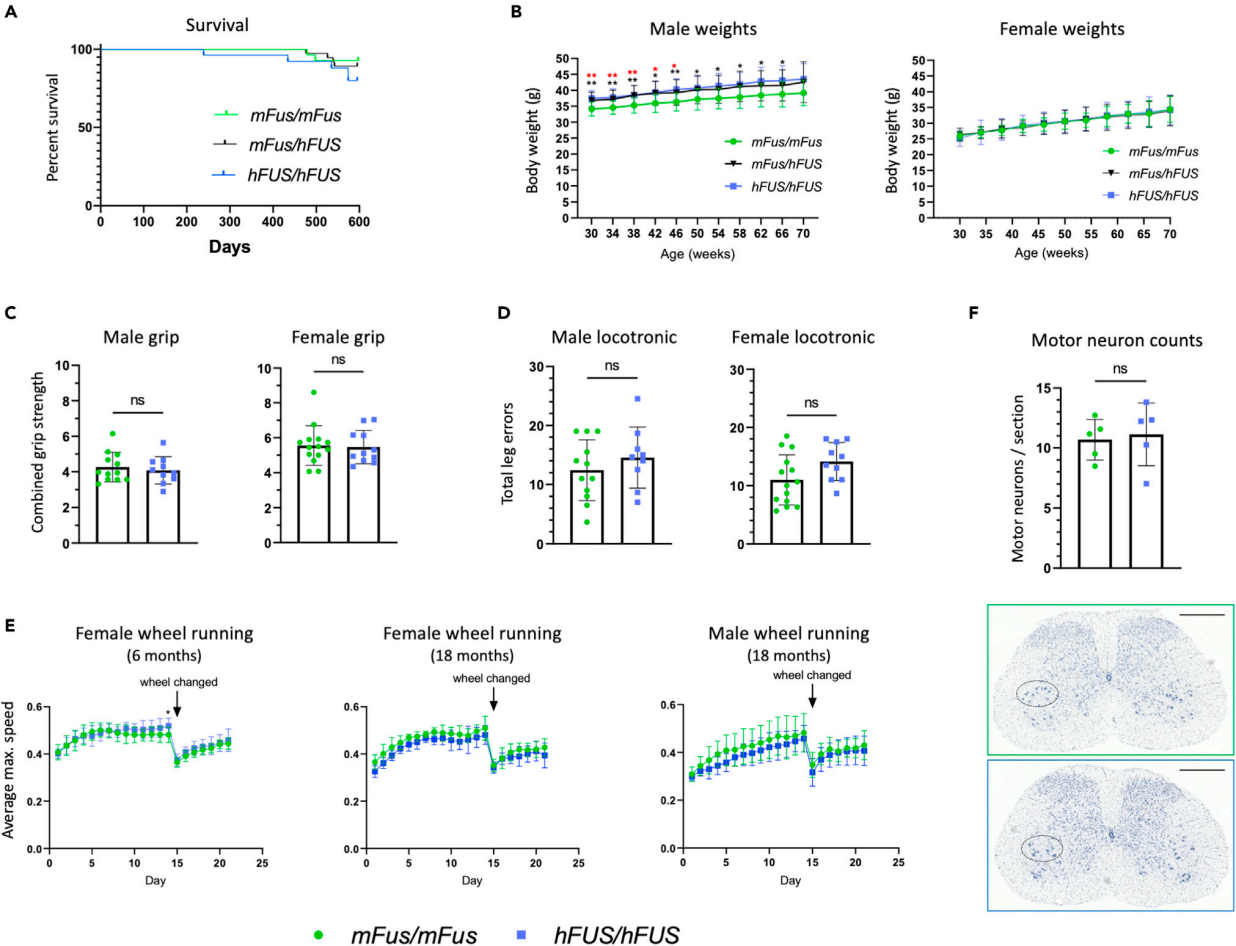
Phenotypic assessments of *hFUS/hFUS* mice show no evidence of an overt phenotype (A) Kaplan–Meier survival curve showing survival data (males and females combined) up to 18 months (n = 33, 47, 30; *mFus/mFus, mFus/hFUS, hFUS/hFUS*). (B) Body weight measurements of male and female humanized FUS mice. *mFus/mFus* (n = 17 male, 16 female), *mFus/hFUS* (n = 22 male, 24 female), *hFUS/hFUS* (n = 14 male, 13 female)*,* up to 16 months of age. Data presented as mean ± SD, ∗ = p ≤ 0.05, ∗∗ = p ≤ 0.01 where black lower∗ is comparing *mFus/mFus* to *hFUS/hFUS* and red upper∗ is comparing *mFus/mFus* to *mFus/hFUS,* calculated using a mixed-effects analysis with Dunnett's post hoc test. (C) Grip strength assessment of *hFUS/hFUS* (n = 10 male, 11 female) and *mFus/mFus* (n = 12 male, 14 female) mice aged 18 months. Data presented as mean ± SD, ns = not statistically significant calculated using Student's t-test. (D) Locotronic testing on *hFUS/hFUS* (n = 9 male, n = 10 female) and *mFus/mFus* (n = 12 male, n = 14 female) mice aged 18 months. Data presented as mean ± SD, ns = not statistically significant calculated using Student's t-test. (E) Motor function via automated wheel running in 6-month-old *hFUS/hFUS* (n = 13 female) and *mFus/mFus* (n = 13 female) mice and 18-month-old *hFUS/hFUS* (n = 6 female, 8 male) and *mFus/mFus* (n = 4 female, 7 male) mice. Data presented as mean ± SD, ∗ = p ≤ 0.05 calculated using a mixed-effects analysis with Šídák's post hoc test. (F) Motor neuron counts in the ventral horn of the lumbar spinal cord in 18-month-old *hFUS/hFUS* and *mFus/mFus* female mice (n = 5 per genotype; representative section images shown below). Scale bar = 0.5 mm. Data presented as mean ± SD, ns = not statistically significant calculated using Student's t-test.

To provide further insight into the impact of humanization, *hFUS* mice were aged to 18 months and studied longitudinally to discern if humanizing the *FUS* gene had any behavioral phenotypic effect, with particular emphasis on motor function. Humanization of the FUS gene had no effect on survival of mice heterozygous or homozygous for human *FUS* as shown by a Kaplan–Meier survival curve (Figure 7A), and homozygous male and female *hFUS* mice were able to breed successfully (data not shown). SHIRPA was carried out as an observational assessment of *hFUS* mouse phenotype of both sexes at 3- and 18-month timepoints (Figure S20). No statistical difference was observed in any of the parameters measured. Body weight measurements show male heterozygous and homozygous *hFUS* mice are marginally, but significantly, heavier than wild-types, on average by 10% across all timepoints measured. Interestingly, this weight difference was not observed in female *hFUS* mice (Figure 7B). Neither sex exhibited motor dysfunction as measured by grip strength nor quantification of leg errors displayed during locotronic analysis (a non-significant trend was observed in locotronic analysis, p = 0.36 males, p = 0.07 females) (Figures 7C and 7D). No deficit in wheel running capacity, including the response to a modified wheel presenting a motor challenge, was observed in *hFUS* mice at early or late timepoints (Figure 7E). Analysis of motor neuron numbers in the sciatic motor pool of the lumbar spinal cord showed no differences between 18-month old *hFUS* and wild-type mice (Figure 7F). Thus, there were no overt phenotypic differences observed between *hFUS* and wild-type mice, consistent with the very minor transcriptomic impact of *Fus* humanization.

## Discussion

ALS/FTD disorders have no cure or effective treatment, and a considerably better understanding of pathomechanisms remains essential for diseases on this spectrum. While cellular and *in vitro* studies are immensely valuable, currently only animal models allow us to look at effects of aging, systemic effects such as hormones or the microbiome, and environmental interactions (De Giorgio et al., 2019). Studies in mice have also shown, for example, that motor neuron death in ALS is not cell autonomous but entails interaction between neurons and other cell types (Taylor et al., 2016). The range of genome engineering techniques now available enables us to tailor mouse models to the research questions that need to be addressed. To optimize mouse models for ALS/FTD research and the development of new therapies, we describe the replacement of whole coding regions, exons and introns included, of three critically important ALS/FTD genes in mouse (*SOD1*, *TARDBP,* and *FUS*), for their human orthologous sequences, creating “genomically humanized” KI mice.

We go on to present the essential deep genomic quality control of these new strains, which we regard as a “new standard” in their development as tools for ALS/FTD research, and we provide some phenotype data as proof of principle for the use of these strains.

Few humanized mice exist and there is no “best approach” to how much of a locus should be knocked in; thus, our strategies were bespoke for each gene based on its genomic context and our current knowledge of pathomechanisms. We have created these genomically humanized KI strains such that the mice express the human splice variants and proteins, noting that on average, human genes express a greater number of splice isoforms than mouse genes (Lee and Rio, 2015), and therefore have increased protein complexity. Furthermore, a small number of amino acid differences within highly conserved mouse and human protein orthologs can significantly affect properties such as propensity to aggregate, for example (Crown et al., 2020; DuVal et al., 2019; Nagano et al., 2015; Perri et al., 2020). Including non-coding gene regions is also important because several known mutations and risk variants exist in such regions (Brown et al., 2021; Dini Modigliani et al., 2014; Ma et al., 2021; Sabatelli et al., 2013). Our genomically humanized strains are “knockin” animals and therefore express proteins from the endogenous locus at physiological levels. Thus, genomic humanization delivers mouse models with human protein isoforms and human protein biochemistry, expressed in an endogenous context, with the potential to model mutations and risk variants in coding or non-coding gene regions (Nair et al., 2019; Zhu et al., 2019). Notably, all three lines survive in homozygosity, immediately showing that for the essential genes *FUS* and *TARDBP*, the human genes can functionally replace their mouse counterpart at the endogenous locus. Aging of hSOD1 (a non-essential gene in mouse) is needed to assess functional equivalency of the human gene expressed endogenously in mouse, although we observe no reduction in weight, which would indicate loss of function (data not shown).

Ensuring locus integrity at the nucleotide level is paramount to understanding the impact of precisely engineered alleles. We demonstrate that targeted long-read sequencing technology, now readily accessible, should be routine for large KI allele quality control in an age of expanding genome engineering capabilities.

In addition to confirming the integrity of our precisely engineered alleles, we took advantage of the strain and sequence differences between the targeting vector and ES cells to map homologous recombination breakpoints. The full length of the long homology arms used, tens of kb in length, were frequently not fully integrated into the locus together with the human gene, suggesting that future strategies may be successful with far shorter regions of homology. Interestingly, we note that for *Tardbp,* breakpoints are closest to the humanized region (11kb 5′ proximal and 5.5kb 3′ distal) and this was the only strain for which we had used CRISPR/Cas9-assisted homology directed repair, with guides cutting specifically within the mouse endogenous *Tardbp* loci. In all strains, we find that recombination breakpoints are free from indels or any other type of mutation.

Looking to the effect on humanized gene products, h*SOD1* expression is not changed in the CNS, but reduced at the mRNA and protein levels only in skeletal muscle. As CNS expression levels are remarkably similar between wild-type and *hSOD1*, this argues against general disruption caused by the conditional element of the allele. One possible explanation for the reduction in skeletal muscle *SOD1* expression could be the presence of tissue-specific transcriptional control elements in the humanized non-coding regions (introns and/or 3′ UTR). Regardless of the mechanism, this finding clearly warrants further investigation. For h*TARDBP*, the minor mRNA expression changes detected did not translate into differences at the protein level or in endogenous TDP-43 splicing function, suggesting that human TDP-43 can functionally replace the mouse protein. Finally, for h*FUS* mice, the moderate reductions in *FUS* mRNA had little consequence at the genome-wide transcriptomic level, showing fewer changes than observed in heterozygous Fus KO tissue, although we cannot rule out more subtle underlying mis-splicing or other unannotated events, which was not possible to analyze with the read depth of this dataset. Overall, the combined lack of detected molecular or behavioral phenotypes functionally corroborates that expression levels detected lie within a physiological range.

These changes underlie the need for characterization of the humanized lines before ALS/FTD mutations are introduced, in order to set a baseline phenotype and provide appropriate humanized-non-mutant controls, while further investigation could lead to insights regarding human-specific gene regulation.

To gain further insight into the impact of humanization, we have more comprehensively characterized *hFUS* mice at the behavioral and molecular level. hFUS homozygotes are fully viable, fertile, and survive as long as wild-type mice through to 18 months of age. We found neither motor phenotypes nor loss of motor neurons in the lumbar spinal cord, in contrast to reports of transgenic hFUS-expressing mice (also without mutations) that show motor phenotypes and motor neuron loss (Ling et al., 2019; Mitchell et al., 2013). While we cannot rule out neurodegenerative phenotypes developing beyond this timeframe, it nevertheless shows remarkable functional synergy with mouse *Fus* and provides a robust foundation for the introduction of pathogenic mutations for future study*.* Looking at body weight data, heterozygous and homozygous *hFUS* males have marginally, but consistently larger body mass than wild-type, which may be related to subtle differences in gene expression levels, but ultimately is a minimal phenotypic difference. Taking phenotypic, transcriptomic, and expression data together, clearly these genomically humanized KI lines are not 100% identical to wild-type animals, which underscores the need to use them as the most accurate genetic control for future mutant lines, in order to distinguish the specific impact of introduced mutations. Further characterization of the h*SOD1* and h*TARDBP* mice at the behavioral and molecular level throughout their entire lifespan will be required to complete their characterization.

These three strains are the first KI lines in which the mouse expresses the human SOD1, TDP-43, and FUS protein from the endogenous locus. These strains will be of great utility for understanding the biology and dysfunction of these proteins when mutated, and is particularly important for the highly dosage-sensitive genes *FUS* and *TARDBP*. The *hTARDBP* strain is the first model to express only human protein without endogenous mouse protein as, to our knowledge, all attempts to rescue the KO phenotype with transgenics (including BACs) have been unsuccessful. In practical terms, our humanized KI lines are significantly simpler to maintain and breed (single locus) versus breeding transgenic animals on a KO background (two loci). While transgenic animals on a KO background can be interbred, this risks genetic drift; in contrast, we regularly refresh our humanized KI lines with C57BL/6J stock, which is relatively straightforward, ensuring the best chance of reproducibility over time.

Furthermore, these humanized models will be an important resource for the validation of ASOs, the highly promising therapeutics aimed at modulating human gene expression levels, as these require the exact human sequence for preclinical trials. Moreover, the humanized mice will uniquely allow us to test the possible effects of ASOs, or any other therapeutic agents, on systemic long-term loss of function that cannot be assessed in transgenic models. This will be critically important as all three genes are ubiquitously expressed and perform crucial biological functions; recent failed clinical trials from ASOs aimed against lowering Huntingtin (*HTT*) showed adverse effects (Kwon, 2021) that might be mediated by *HTT* loss of function, highlighting the growing need for improved preclinical understanding of the risks associated with long-term gene therapies (Editorial, 2021).

We envisage that these mice, which will be freely available from the European Mutant Mouse Archive (EMMA), may be mutated by CRISPR or other approaches to recreate human ALS/FTD causal mutations, to dissect pathogenesis including aggregation, and for developing new therapies, and we encourage their uptake by the community.

### Limitations of the study

We need a variety of models to study the complexities of ALS and FTD. Despite the limitations of the mouse as a system to model human neurodegenerative disease (including differences in axon length, rate of aging, synaptic connections between upper and lower motor neurons, and anatomy of neuromuscular junctions), mice will continue to be invaluable to study fundamental disease processes. They provide a complex *in vivo* environment that is critical for the study of interactions between multiple tissues and cell types (motor neurons, glia, muscle, and other non-neuronal tissues) that are likely necessary for disease development and progression. By generating these humanized mice for ALS/FTD research, we aim to produce, at least at the biochemical level, the closest models to the human condition to study the impact of human genes and proteins within a complex mammalian organism. These new humanized models are not designed to entirely replace existing models. Physiological models for ALS/FTD (i.e. those that involve study of genes at endogenous loci), typically have slow onset of disease and may not reach end-stage; therefore, to address questions surrounding late or end-stage disease, transgenic overexpression models may be better suited.

In general, a potential critical limitation of genomically humanized mice is that replacing the endogenous mouse gene by its human *wild-type* counterpart could lead to functional consequences. As a proof of principle, we present an extensive characterization of h*FUS* mice showing that although minimal, some differences exist between h*FUS* mice and littermate controls. Taking phenotypic, transcriptomic, and expression data together, clearly these lines are not identical to wild-type animals, which underscores the need to use them as the most accurate genetic control for future mutant lines, in order to assess the impact of mutations specifically, distinct from the impact of humanization. Further characterization of the h*SOD1* and h*TARDBP* mice at the behavioral and molecular level throughout their entire lifespan will also be required to complete their characterization. Moreover, humanized mice (as with other mutant mouse strains) will require continued quality control to make sure that the human allele remains intact through the process of colony maintenance.

With the three humanized lines, we decided to maintain the mouse promotor and upstream non-coding exons. The rationale for this approach was to minimize disruption of expression from the endogenous locus within the context of the mouse. Thus, these models will not allow us to study the possible effects on expression of the human promoter regions (or variants within), although we note further modifications can be made to the wild-type humanized animals, which are essentially templates for further changes.

Looking to the future, single humanized alleles may not be sufficient to study disease mechanisms involving binding partners of humanized proteins, or to assess human-specific splicing events (Brown et al., 2021; Ma et al., 2021), and new bespoke humanized alleles may need to be engineered and crossbred for these purposes. In turn, these may aid in more faithfully recapitulating later stage pathology and phenotypes, for further mechanistic insight *in vivo* and for improved read outs for testing therapeutic strategies. The three humanized alleles presented here are an important first step and proof of principle that genomically replacing mouse endogenous ALS/FTD genes with their human counterparts can functionally replace their mouse orthologs, enabling the introduction of mutations to better understand the impact of human mutant or variant genes and proteins in a physiological context.

## STAR★Methods

### Key resources table

**Table:** 

REAGENT or RESOURCE	SOURCE	IDENTIFIER
**Antibodies**		

Anti TDP-43 (pan)	Bio techne	MAB7778
Anti TDP-43 (human specific)	Proteintech	60019-2-Ig; RRID:AB_2200520
Anti TDP-43 (pan)	Abcam	ab104223; RRID:AB_10710019
GAPDH	ThermoFisher	AM4300; RRID:AB_2536381
GAPDH	Proteintech	60004-1-Ig; RRID:AB_2107436
FUS 562	Novus	NB100-562; RRID:AB_10002858
FUS 565	Novus	NB100-565; RRID:AB_2105207
Anti SOD1 (pan)	Professor Stefan Marklund, Medical Biosciences, Umeå University, Sweden	N/A
Anti SOD1 (pan, native protein conformation)	Professor Stefan Marklund, Medical Biosciences, Umeå University, Sweden	N/A
IRDye 800CW Goat anti-Rabbit IgG (H+L)	LI-COR	926-32211; RRID:AB_621843
SOD1	Professor Stefan Marklund, Medical Biosciences, Umeå University, Sweden	N/A

**Chemicals, peptides, and recombinant proteins**		

Trizol reagent	Life technologies	15596018
RIPA buffer	ThermoFisher	89900
cOmplete Mini Protease Inhibitor	Roche/Sigma	4693124001
Prolong Glass Antifade Mountant with NucBlue	ThermoFisher	P36981
Gallocyanine	Sigma-Aldrich	124508-10G
CV Ultra Mounting Media	Leica	14070936261

**Critical commercial assays**		

Mouse Tardbp qPCR Taqman assay Mm00735064	ThermoFisher	Mm00735064_cn
Human TARDBP qPCR Taqman assay Hs06560655	ThermoFisher	Hs06560655_cn
RNeasy Lipid Tissue Mini Kit	Qiagen	74804
RNeasy Fibrous Tissue Mini Kit	Qiagen	74704
MultiScribe Reverse Transcriptase	ThermoFisher	4311235
High Capacity cDNA Reverse Transcription Kit	Applied biosystems	4368814
qScript cDNA Synthesis	Quanta Bio	95047-500
qPCR Bio SyGreen mix LO-ROX	PCR Biosystems	PB20.11-05
Dream taq green PCR master mix (2X)	Thermo Scientific	K1081
Fast SYBR Green Master Mix	ThermoFisher	4385610
DC Protein Assay	BioRad	500-0116
REVERT Total Protein Stain Kit	LI-COR	926-11010

**Deposited data**		

RNAseq fastq files deposited to NCBI Sequence Read Archive (SRA)	https://www.ncbi.nlm.nih.gov/sra/PRJNA769952	SRA: PRJNA769952

**Experimental models: Cell lines**		

Mouse embryonic stem cell line R1	https://web.expasy.org/cellosaurus/CVCL_2167	RRID:CVCL_2167

**Experimental models: Organisms/strains**		

Humanised SOD1 mice: Sod1^tm1.1(SOD1)Emcf^	This paper	MGI:6403195; EM:13075
Humanised TARDBP mice: Tardbp^em2.1(TARDBP)H^	This paper	MGI:6513996, EM:14603
Humanised FUS mice: Fus^tm3.1(FUS)Emcf^	This paper	MGI:6193752; EM:13073
Flpo expressing mouse strain: Gt(ROSA)26Sortm2(CAG-flpo,-EYFP)Ics	Birling et al., 2012	MGI:5285396

**Oligonucleotides**		

Primers	See Table S1	N/A

**Recombinant DNA**		

Mouse Sod1 BAC	https://resourcedb.nbrp.jp/	B6Ng01-068O19
Human SOD1 BAC	bacpacresources.org	BAC RP11-535E10
Mouse Tardbp BAC	https://resourcedb.nbrp.jp/	B6Ng01-103M13
Human TARDBP BAC	bacpacresources.org	RP11-829B14
Mouse Fus BAC	bacpacresources.org	RP24-297F14
Human FUS BAC	bacpacresources.org	RP11-157F22
Humanised SOD1 targeting BAC	This paper	N/A
Humanised TARDBP targeting BAC	This paper	N/A
Humanised FUS targeting BAC	This paper	N/A
pSpCas9(BB)-2A-Puro (PX459)	Addgene (Ran et al., 2013)	RRID:Addgene_62988

**Software and algorithms**		

Minimap2	Li, 2018	github.com/lh3/minimap2
NGMLR	Sedlazeck et al., 2018	github.com/philres/ngmlr
Samtools	Li et al., 2009	github.com/samtools/
Integrated Genomics Viewer	https://software.broadinstitute.org/software/igv/	N/A
STAR aligner	Dobin et al., 2013	github.com/alexdobin/STAR
DEseq2	Love et al., 2014	github.com/mikelove/DESeq2
Graphpad Prism	Graphpad software (CA, USA)	N/A
ImageJ	National Institutes of Health	N/A
ilastik	Berg et al., 2019	www.ilastik.org

**Other**		

Grip strength meter	Bioseb	N/A
Locotronic system	Intellibio	N/A
Automated wheel-running system	TSE systems	N/A

### Resource availability

#### Lead contact

Further information and requests for resources and reagents should be directed to and will be fulfilled by the lead contact, Thomas J. Cunningham (t.cunningham@har.mrc.ac.uk).

#### Materials availability

• BAC targeting constructs generated in this study are available upon request with a completed Materials Transfer Agreement.
• Mouse lines generated in this study are available from the European Mouse Mutant Archive (EMMA). *hSOD1* (MGI:6403195; EM:13075), *hTARDBP* (MGI:6513996, EM:14603), *hFUS* (MGI:6193752; EM:13073).

### Experimental model and subject details

#### Mice

All mice were maintained and studied according to UK Home Office legislation at MRC Harwell (Home Office Project Licence 20/0005), with local ethical approval (MRC Harwell AWERB committee). Mice were fed *ad libitum* (Rat and Mouse Breeding 3 (RM3), Special Diet Services) with free access to water (chlorinated to 9-13 ppm). Mice were kept under constant conditions with a regular 12 h light (19:00-07:00) and dark (07:00-19:00) cycle, temperature of 21±2°C and humidity 55±10%. Mice were housed in same sex cages of up to five mice. Cages contained Aspen Chips bedding (Datesand), shredded paper and a rodent tunnel for enrichment. All three humanised mice strains are maintained by backcrossing humanised heterozygous animals to C57BL/6J mice. For all the experimental cohorts presented, mice were obtained by intercrossing heterozygous humanised animals to obtain all three genotypes within a single cross, with randomised weaning into cages. Males and females were used for *hSOD1* and *hTARDBP* mice expression analyses, respectively, with longditudinal analyses in both sexes ongoing. Both males and females were used for longitudinal analyses of *hFUS* mice.

##### Genomically humanised *SOD1* mice

The official allele designation is *Sod1*^tm1.1(SOD1)Emcf^ (MGI:6403195), referred to here as *hSOD1*. A BAC targeting construct harbouring the mouse *Sod1* locus (BAC B6Ng01-068O19; strain C57BL/6N) was engineered to replace the mouse *Sod1* gene from the ATG start site (g.117) to the end of the 3’UTR (g.5583) with the orthologous human *SOD1* genomic sequence from a BAC harbouring the human *SOD1* locus (BAC RP11-535E10). *LoxP* sites were inserted into intron 3, and 1kb downstream of the inserted human 3’UTR (Figure 1A). The sequence between the *loxP* sites was duplicated to allow conditional expression of a second copy of the sequence. An *FRT*-flanked Neomycin cassette was inserted between the first and second copies of the duplicated sequence. This construct was electroporated into the 129X1/SvJ-129S1/SV mouse ES cell line R1, humanising *Sod1* in the mouse genome via homologous recombination. Correctly targeted clones were initially identified through Loss-Of-Allele (LOA) copy via qPCR and ddPCR assays, while karyotype of positive clones was then screened employing ddPCR (Codner et al., 2016; Valenzuela et al., 2003). Mice were generated by injection of modified ES Cells into donor blastocysts, with the resultant chimeric male offspring crossed to C57BL/6J females to obtain germline transmission (GLT). Following confirmation of GLT, *hSOD1* heterozygous mice were backcrossed for one further generation to the C57BL/6J strain. Selection cassette removal was performed at generation N3 C57BL/6J backcrossed animals, via cytoplasmic injection of IVF derived 1-cell embryos with *Flpo* mRNA. The Neo-negative line was then backcrossed for one further generation onto C57BL/6J, followed by intercrossing to produce animals at generation N4 for long-read sequencing and preliminary expression analysis. Genotyping was performed by LOA copy number qPCR using custom probe sets: Mouse *Sod1* allele*,* forward primer GTACCAGTGCAGGACCTCAT, reverse primer AGCGTGCTGCTCACCTCT, 5’-FAM probe AACATGGTGGCCCGGCGGATG; *hSOD1* allele, forward primer GTGCAGGTCCTCACTTTAATCC, reverse primer CCAGAAAGCTATCGCCATTATTACAAG, 5’-FAM probe CCAAAGGATGAAGAGAGGTAACAAGATGC. This line is available from the European Mouse Mutant Archive (EMMA, EM:13075).

##### Genomically humanised *TARDBP* mice

The official allele designation is *Tardbp*^em2.1(TARDBP)H^ (MGI:6513996), referred to here as *hTARDBP.* A BAC targeting construct harbouring the mouse *Tardbp* locus (BAC B6Ng01-103M13; strain C57BL/6N) was engineered to replace the mouse *Tardbp* gene from the ATG start site to the TAG stop codon with the orthologous human *TARDBP* genomic sequence from a BAC harbouring the human *TARDBP* locus (BAC RP11-829B14); an *FRT*-flanked Neomycin cassette was inserted into intron 3 of the gene (Figure 2A). This construct was electroporated into the 129X1/SvJ-129S1/SV mouse ES cell line R1, humanising *Tardbp* in the mouse genome via homologous recombination assisted by CRISPR-Cas9 (delivered by co-electroporation with px459 plasmids (Ran et al., 2013); sgRNA guide insert sequences: gCTCCACCCATATTACCACC and gGTCGGGCCCATCTGGGAATA). Correctly targeted clones were initially identified through Loss-Of-Allele (LOA) copy via qPCR and ddPCR assays, while karyotype of positive clones was then screened employing ddPCR (Codner et al., 2016; Valenzuela et al., 2003). Mice were generated by injection of modified ES Cells into donor blastocysts, with the resultant chimeric male offspring crossed to C57BL/6J females to obtain GLT. Following confirmation of GLT, *hTARDBP* heterozygous mice were bred one further generation to the C57BL/6J strain, followed by intercrossing to generate homozygotes for long-read sequencing and preliminary expression analysis. Cohorts used for analysis here still contain the selection cassette. Genotyping was performed by Loss-Of-Allele (LOA) copy number qPCR using the commercially available TaqMan probesets Mm00735064 (mouse) and Hs06560655 (human) (ThermoFisher). This line has been submitted to the European Mouse Mutant Archive (EMMA, EM:14603).

##### Genomically humanised FUS mice

The official allele designation is Fus^tm3.1(FUS)Emcf^ (MGI:6193752), referred to here as *hFUS*. A BAC targeting construct harbouring the mouse *Fus* locus (BAC RP24-297F14; strain C57BL/6J) was engineered to replace the mouse *Fus* gene from the ATG start site (g.106) to the end of the 3’UTR (g.14574) with the orthologous human *FUS* genomic sequence from a BAC harbouring the human *FUS* locus (BAC RP11-157F22), and an *FRT*-flanked Neo cassette was inserted 1kb downstream from the humanised 3’UTR (Figure 3A). This construct was electroporated into the 129X1/SvJ-129S1/SV mouse ES cell line R1, humanising *Fus* in the mouse genome via homologous recombination. Correctly targeted clones were initially identified through Loss-Of-Allele (LOA) copy via qPCR and ddPCR assays, while karyotype of positive clones was then screened employing ddPCR (Codner et al., 2016; Valenzuela et al., 2003). Mice were generated by injection of modified ES cells into donor blastocysts, with the resultant chimeric male offspring crossed to C57BL/6J females to obtain GLT. Following confirmation of GLT, *hFUS* heterozygous mice were bred one further generation to the C57BL/6J strain, followed by crossing to the Gt(ROSA)26Sortm2(CAG-flpo,-EYFP)Ics line (C57BL/6Ntac background) (Birling et al., 2012) to excise the *FRT*-flanked Neo selection cassette. The Neo-negative line was then backcrossed for at least six further generations onto the C57BL6/J background before homozygotes were generated for long-read sequencing and experimental cohorts were bred. Genotyping was performed by LOA copy number qPCR using custom probe sets: Mouse *Fus* allele*,* forward primer GGCGGTTGTGTGTGTATGTG, reverse primer AACATGGACCCATTCTTCAGAAAG, 5’-FAM probe CATCATTTTAGTTAAATTCTGTTTCC; *hFUS* allele, forward primer CCCAGCAGGAACTGGAATACAG, reverse primer AACATGGACCCATTCTTCAGAAAG, 5’-FAM probe TTCTGTCATGGGGAAATTCTGTTTCCC. This line is available from the European Mouse Mutant Archive (EMMA, EM:13073).

### Method details

#### Allele verification pipeline

##### Xdrop enrichment, Oxford Nanopore sequencing and analysis

Xdrop^TM^ enrichment, amplification, and Oxford Nanopore sequencing was performed by Samplix Services (Denmark) (Blondal et al., 2021) (Figure S1). High molecular weight DNA was extracted from homozygous humanised mouse tissue using SDS/proteinase K lysis buffer (100 mM NaCl, 10 mM Tris-Cl pH 8, 25 mM ETDA pH 8, 0.5% SDS, 20 μg/ml RNAse A, 100 μg/ml proteinase K) and phenol/chloroform purification (gentle mixing/inversion only, no vortexing), followed ethanol precipitation and elution in 10 mM Tris-Cl pH 8.5. DNA was evaluated by Tapestation 2200 System (Agilent Technologies Inc.), using Genomic DNA ScreenTape according to the manufacturer’s instructions (average sizes: *hSOD1*>100 kb, *hTARDBP*>30 kb, *hFUS*>60 kb). A series of ‘Detection Sequence’ droplet PCR (dPCR) assays, and accompanying qPCR validation assays in the adjoining region, were designed to span each locus (Table S1; Figures S2–S4). Primer efficiency of the assays was tested by serial sample dilution in Samplix Primer test PCR kit according to manufacturer’s instructions. 8-9 ng DNA was partitioned in droplets by Xdrop™ and subjected to dPCR using the Detection Sequence assay. The droplet productions were then stained and sorted by fluorescence-activated cell sorting (FACS). DNA was then released from the isolated droplets, and DNA was again partitioned in droplets by Xdrop™ and amplified by droplet multiple displacement amplification (dMDA) as previously described (Blondal et al., 2021). After amplification, DNA was isolated and quantified, and enrichment was validated by qPCR (Data S1) before Oxford Nanopore Sequencing.

Minion Oxford Nanopore Sequencing libraries were prepared from the dMDA samples as described by the manufacturer’s instructions for Premium whole genome amplification protocol (SQK-LSK109) with the Native Barcoding Expansion 1-12 (EXP-NBD104) including a T7 endonuclease I digestion step for debranching followed by bead purification (MagBio). Generated raw data (FAST5) was subjected to base-calling using Guppy v.3.4.5 with high accuracy and quality filtering to generate FASTQ sequencing data (Samplix Services). 3-5 Gb of sequencing data was obtained for each sample. Subsequently, the data was aligned with minimap2 (Li, 2018) to the mouse reference C57BL/6J genome (GRCm38.p6), the mouse reference strain 129 genome (129S1_SvImJ), the human reference genome (GRCh38.p13), and custom reference sequences representing the intended allele sequences. In addition, the *hTARDBP* sample was aligned using NGMLR (Sedlazeck et al., 2018). Samtools (Li et al., 2009) was used to convert, sort, and index alignment data files for visualization in IGV (Robinson et al., 2011). Bioinformatics commands for the alignments can be found in Figure S21.

#### Gene expression

##### Tissue isolation

Mice were culled via intraperitoneal injection of pentobarbitone and extracted tissues were stored at -80^o^C until processed. Lumbar spinal cord tissue for staining was collected by hydraulic extrusion of the whole spinal cord into cold PBS. The lumbar region was identified visually, cut, and embedded in OCT and frozen over isopentane and dry ice.

##### RT-PCR and Quantitative real-time PCR

Total RNA was isolated from frozen tissue using RNeasy lipid or fibrous tissue mini kits (Qiagen) or Trizol reagent (ThermoFisher) following manufacturer’s instructions. cDNA was synthesised using MultiScribe Reverse Transcriptase kit (ThermoFisher) or qScript cDNA Synthesis kit (QuantaBio) following manufacturer’s instructions. For RT-PCR analysis of humanised and mouse transcripts, human- and mouse-specific primer pairs were designed for each gene (Table S1). PCR reaction products were run on 1.5% agarose gels to assess for the presence/absence of mouse or human gene products.

To quantitatively analyse mRNA expression, and for *FUS* splicing assays, qRT-PCR was performed using Fast SYBR Green Master Mix (ThermoFisher) or MasterMix qPCR Lo-ROX (PCR-Bio) and 200 nM or 250 nM of each forward and reverse primer. The thermal amplification conditions were: 95°C for 20 s, then 40 cycles of 95°C for 3 s and 60°C for 30 s. The specificity of primer binding and amplification was confirmed by melt curve analysis. To control for non-specific amplification, no-template reactions were performed using all reagents except the sample. qRT-PCR primer pairs are listed in Table S1. qRT-PCR reactions were carried out on 7500, QuantStudio or Roche Lightcycler 480 II Real-Time PCR machines. qRT-PCR data was analysed using the manufacturer’s analysis software following the 2^-ΔCt^ method.

##### Protein expression analysis

For FUS and SOD1, tissue was disrupted in Pierce RIPA buffer (ThermoFisher) + protease inhibitors (Roche/Sigma), whereas TDP-43 was disrupted in Urea 7M-1% SDS, followed by matrix D lysing tubes for 2x 30 s at 5500 rpm (Precellys). Homogenates were centrifuged at 13,000x g for 10 minutes at 4°C. Supernatant (total protein) was aliquoted and stored at -80°C. Genotypes studied included wildtype, heterozygous and homozygous mice; at least n=3 per genotype for different tissues, including: brain, spinal cord, skeletal muscle and liver; except for spinal cord and TA for *TARDBP* humanised mice where n=2 was used. Protein concentration was assessed using a DC assay (BioRad) or BCA assay (Sigma). FUS protein extracts were ran on 4-12% Bis-Tris protein gels (NuPAGE, ThermoFisher) with 1X MOPS (ThermoFisher) + 500 μl antioxidant (ThermoFisher) at 200V. SOD1 protein extracts were ran on Novex 14% Tris-Glycine protein gels (ThermoFisher) with 1X Tris-Glycine SDS (ThermoFisher) at 200V. FUS and SOD1 protein was transferred to a nitrocellulose membrane using iBlot Transfer Stacks and iBlot machine. For TDP-43, protein extracts were run on 10% Bis-Tris gels with Tris-Glycine SDS running buffer at 150V, followed by wet transfer with running buffer and 20% methanol at 280 mA. For SOD1, membranes were reversibly stained for total protein using a Revert 700 Total Protein Stain Kit (LI-COR) following the manufacturer’s instructions. After incubation with 4% skimmed milk (Sigma Aldrich) in PBST (0.1% Tween-20) for 1.5 h, membranes were incubated with the following antibodies: FUS 562 (a rabbit polyclonal raised against a peptide matching the C-terminus of both mouse and human FUS; Novus NB100-562, 1:10,000), pan TDP-43 (a mouse monoclonal antibody recognising an epitope of the N-terminus of TDP-43 that is identical between mouse and human, Bio techne, MAB7778, 1:1000), human specific TDP43 (a mouse monoclonal against the C-terminus of TDP-43 that recognises human TDP-43 protein but not mouse TDP-43, Proteintech 60019-2-Ig, 1:1000), pan SOD1 (a custom-made goat polyclonal antibody recognising a C-terminal epitope of SOD1 that is identical between mouse and human proteins; 1:1000; kind gift from Professor Stefan Marklund, Umea University, Sweden), and GAPDH (ThermoFisher AM4300, 1:2000 or Proteintech 60004-1-Ig, 1:1000) diluted in 4% milk/PBST either O/N at 4°C or for 1.5 h at RT. Membranes were washed 3x 5 minutes in PBST then incubated with anti-rabbit and anti-mouse antibodies (LI-COR, 1:15,000 or ThermoFisher, 1:10,000) diluted in 4% milk/PBST for 1.5 h at RT. Membranes were washed 2x 5 minutes in PBST and a further 5 minutes in PBS before being dried (10 minutes) and imaged on a LI-COR Odyssey Scanner or an Image Quant LAS4000, GE healthcare. Blots were quantified using Empiria Studio 1.3 (LI-COR) or image count TL imaging software (GE healthcare). Blot images from *hFUS* samples are shown in Figure S18.

#### Immunohistochemistry of motor cortex and spinal cord and image analysis

*hFUS* brain (8 months) was dissected into left and right sides and again into anterior and posterior portions, drop fixed in 4% paraformaldehyde (PFA) for 24 hours, washed in PBS 3 x 10 minutes, equilibrated in 30% sucrose, and cryopreserved in OCT over isopentane on dry ice. Serial coronal sections (20 μm) were cut through the motor cortex region of the anterior embedded brain tissue on a cryostat. hFUS (18 months) spinal cords were collected by hydraulic extrusion and the lumbar region identified, dissected, and frozen in OCT over isopentane on dry ice. Serial transverse sections (12 μm) were cut on the cryostat and stored at -20°C. For FUS staining, sections were post-fixed in 4% PFA for 10 minutes; washed 3x 10 minutes in PBS then incubated in blocking buffer (10% normal goat serum in TBST, 0.2% Triton) for 1 h; washed 3x 10 minutes in PBS then incubated with an anti-FUS 565 antibody (NB100-565; 1:500, Novus) in blocking buffer overnight at 4^o^C; washed 3x 10 minutes in PBS; incubated with anti-mouse IgG (1:1500, AlexaFluor 488, ThermoFisher) in blocking buffer for 2 h; washed 3x 10 minutes in PBS; and mounted using Prolong Glass Antifade Mountant with NucBlue for nuclear counterstaining (ThermoFisher). Snap images were obtained using an inverted confocal microscope (Zeiss).

*hSOD1* lumbar spinal cords (6 months) were dissected and embedded in OCT over isopentane pre-cooled in liquid nitrogen. Serial transverse sections (12 μm) were cut on a cryostat. Sections were washed 3x 10 minutes in PBS; incubated in blocking buffer (2% bovine serum albumin in TBST, 0.1% Triton) for 1 h; incubated with native pan-SOD1 antibody (antibody recognising an epitope of native SOD1 that is identical between mouse and human proteins, kind gift from Professor Stefan Marklund, Umea University, Sweden, 1:1000) in blocking buffer overnight at 4^o^C; washed 3x 10 minutes in PBS; incubated with anti-goat IgG (1:1500, AlexaFluor 488, ThermoFisher) in blocking buffer for 1 h; washed 3x 10 minutes in PBS; and mounted using Prolong Glass Antifade Mountant with NucBlue (ThermoFisher). Z-stack images were obtained using an inverted confocal microscope (Zeiss).

For *hTARDBP*, 10-week old mice were transcardially perfused with 4% PFA, and the brain dissected followed by equilibration in 30% sucrose and cryopreserved in OCT at -20°C. Serial coronal cuts (20 μm) around the bregma were made and collected onto glass slides. Slides were permeabilized using antigen retrieval (10mM Sodium Citrate, 0.05% Tween 20, ph 6.0) at 85°C for 20 minutes; washed in 0.1% Triton X-100 in PBS for 20 minutes; blocked with 1% BSA and Normal goat serum for 1 h; incubated overnight at 4°C with primary antibody against TDP-43 (1:1000, Abcam, ab104223) in blocking buffer; washed and incubated with secondary antibody (Alexa Fluor 488 (Thermo Fisher)). Slides were mounted with Mowiol reagent (Millipore) and DAPI and images captured using a Zeiss LSM 510 confocal microscope with a 63x oil objective.

#### Determination of muscle fibre size and central nucleation

Tibialis anterior muscles from *hSOD1* (4 months) and *hFUS* (18 months) mice were embedded in OCT and flash frozen over isopentane pre-cooled in liquid nitrogen. 8μm muscle sections were cut, stained with hematoxylin and eosin, and digitized using a slide scanner (Zeiss axioscan Z1). For each animal, three 500 μm x 500 μm areas of muscle tissue were selected for determination of muscle fibre size. Muscle fibre segmentation was performed in ilastik software (Berg et al., 2019) and ImageJ (National Institutes of Health), and checked manually. The minimum feret diameter for each fibre was calculated in ImageJ. Central nucleation was manually assessed in 500 cells per muscle and expressed as a percentage.

#### RNA sequencing

Whole spinal cords were extracted via hydraulic extrusion of 3-month old male animals and stored in RNAlater (ThermoFisher); genotypes included wildtype, *hFUS* homozygotes, and *Fus* KO heterozygotes; n=4 per genotype. Total RNA extraction, library preparation, Illumina sequencing, and bioinformatics analysis was performed by Lexogen services. Total RNA extraction using Lexogen SPLIT kit; Library preparation using CORALL Total RNA-Seq Library Prep Kit with Poly(A) selection; SR75 High Output sequencing on Illumina NextSeq 500. Data was analysed using the Lexogen CORALL Data Analysis Pipeline, including sequence QC (fastqc), read trimming (cutadapt), mapping, quantification, and differential expression. FASTQ files of raw reads were mapped to the GRCm38 mouse genome using STAR (Dobin et al., 2013). Gene and transcript expression were estimated with Lexogen’s Mix2 software. Transcript level differential expression was performed using DESeq2 (Love et al., 2014). More detail can be found in Data S1.

#### SNP analysis

Transnetyx services performed MiniMUGA SNP analysis; an array-based platform with over 10,000 SNP markers that can be used to determine genetic background in 241 inbred strains of mice.

#### Phenotyping

Phenotyping tests on *hFUS* mice were carried out at the same time each day and mice were acclimatised for 30 minutes prior to each test. The same experimenter carried out each test and was blind to genotypes of the mice.

##### SHIRPA

Modified SHIRPA (Rogers et al., 1997) was carried out at 3 months and 18 months; 3 months: n=17,15,16,13 male *mFus/mFus,* male *hFUS/hFUS,* female *mFus/mFus,* female *hFUS/hFUS,* 18 months: n=12,10,14,11 male *mFus/mFus,* male *hFUS/hFUS,* female *mFus/mFus,* female *hFUS/hFUS*). Any significant differences observed between genotypes for SHIRPA parameters were assessed using Fisher’s exact test and chi-square tests.

##### Weight checks

Mice were weighed inside a beaker, without the use of anaesthetic, every four weeks and before relevant *in vivo* tests.

##### Grip strength

Grip strength of 22 males (n=12 wildtype and n=10 *hFUS* homozygous mice) and 25 females (n=14 wildtype and n=11 *hFUS* homozygous mice) aged 18 months was assessed using the force sensor equipment as per manufacturer’s instructions (Bioseb Grip Strength Meter). Mice were lowered onto the grid so only the forelimbs could grip, and were pulled steadily downward. This was repeated 3 times. Mice were lowered onto the grid allowing fore- and hind limbs to grip, and were pulled steadily downward. This was repeated 3 times.

##### Locotronic

Locotronic foot misplacement analysis of 22 males (n=12 wildtype and n=10 *hFUS* homozygous mice) and 25 females (n=14 wildtype and n=11 *hFUS* homozygous mice) aged 18 months was assessed as per manufacturer’s instructions (Intellibio). The equipment contains a horizontal ladder within a corridor and mice are motivated to move from the illuminated start area to the darker finish area. Infrared sensors above and below the ladder detect errors in paw placement. Three runs were carried out per mouse with at least 30 minutes between runs, and the mean average total leg errors calculated. Runs which took longer than 30 s after exiting the start area were discounted (Stewart et al., 2019).

##### Wheel-running motor function (MO)

Motor function of 26 female mice (n=13 wildtype and n=13 *hFUS* homozygous mice) aged 6 months and 18 males (n=9 wildtype and n=9 *hFUS* homozygous mice) and 18 females (n=9 wildtype and n=9 hFUS homozygous mice) aged 18 months was assessed using an automated wheel-running test (Mandillo et al., 2014). Mice were singly housed in cages containing a voluntary running wheel attached to a computer for recording data for three weeks. Food and water were available *ad libitum*. After two weeks, the wheel was changed for one with rungs missing at irregular intervals, presenting a motor challenge. Only data recorded during the dark period (19:00-07:00) was analysed. Average maximum wheel running speed was calculated (in 5-minute bins) and analysed. Any mice which did not complete the three weeks were removed from analysis; in particular, aged mice are more prone to weight loss with this test, which is cause for removal before they reach humane endpoints.

##### Motor neuron counts in lumbar spinal cord

5 female *mFus/mFus*, and 5 female *hFUS/hFUS* mice were sacrificed at 18 months, spinal cord was removed via hydraulic extrusion, and a 1 cm section of the lumbar spinal cord bulge (centring on the widest point of the lumbar enlargement) was dissected and embedded in OCT and frozen over isopentane on dry ice. Serial transverse sections (20 μm) were cut from regions L1 to L6 of the lumbar spinal cord and collected onto glass slides. Every third section was analysed leaving a gap of 60 μm between sections and ensuring the same motor neuron was not counted twice. Slides were stained for 20 minutes in Gallocyanin (0.3 g gallyocyanin, 10 g chrome alum, distilled water up to 100 ml), rinsed with water, dehydrated and mounted using CV Ultra Mounting Media (Leica Biosystems). Sections were scanned using a Nanozoomer slide scanner (Hamamatsu) and motor neurons counted. Motor neurons with the following criteria were counted: a dense, visible nucleolus, a diameter of >15 μm and visible dendritic branching. 35-40 sections were counted per animal and the level of the spinal cord standardised by morphological assessment, such that equivalent sections from L1-L6 were included for counting, centring on L4-L5.

### Quantification and statistical analysis

Statistical analysis was conducted using GraphPad Prism and SPSS. Two groups were compared with a single time point using Student’s t-test. Two groups were compared across multiple time points using 2 way ANOVA with Šídák's multiple comparisons post hoc test. Three or more groups were compared at a single time point using one-way ANOVA with Dunnett’s post hoc test. Repeated measures data was analysed using a Restricted Maximum Likelihood (REML) linear mixed model due to random missing values; as the values are missing at random the results can be interpreted like a repeated measures ANOVA. SHIRPA data was analysed using Fisher’s exact and chi square tests. Statistical analysis of qRT-PCR data was performed on ΔCT values. Statistical significance was defined as p ≤ 0.05 for analysis of phenotyping and molecular biology data, and padj < 0.1 for analysis of differentially expressed transcripts in RNAseq data (statistical values for the latter generated with DEseq2). Refer to figure legends for n numbers; n numbers refer to biological replicates (i.e. number of animals used in animal experiments). Statistical detail for each experiment can be found in the figure legends. Where indicated, ∗=p≤0.05, ∗∗=p≤0.01, ∗∗∗=p≤0.001, ∗∗∗∗=p≤0.0001.

## Data Availability

• RNA-seq data have been deposited at NCBI Sequence Read Archive (SRA) and are publicly available. Accession numbers are listed in the key resources table. • This paper does not use original code. • Any additional information required to reanalyze the data reported in this paper is available from the lead contact upon request.

## Supplemental Information

Document S1. Figures S1–S21

Table S1. Primer and probe sequences

Data S1. Samplix qPCR enrichment data, SNP profiling data and RNAseq details, relating to STAR Methods, and Figure 6
