# Generation and analysis of innovative genomically humanized knockin *SOD1*, *TARDBP* (TDP-43), and *FUS* mouse models

**DOI:** 10.1016/j.isci.2022.103999

**Published:** 2022-03-12

**Authors:** Anny Devoy, Georgia Price, Francesca De Giorgio, Rosie Bunton-Stasyshyn, David Thompson, Samanta Gasco, Alasdair Allan, Gemma F. Codner, Remya R. Nair, Charlotte Tibbit, Ross McLeod, Zeinab Ali, Judith Noda, Alessandro Marrero-Gagliardi, José M. Brito-Armas, Chloe Williams, Muhammet M. Öztürk, Michelle Simon, Edward O’Neill, Sam Bryce-Smith, Jackie Harrison, Gemma Atkins, Silvia Corrochano, Michelle Stewart, Jonathan D. Gilthorpe, Lydia Teboul, Abraham Acevedo-Arozena, Elizabeth M.C. Fisher, Thomas J. Cunningham

## Main text

(iScience *24*, 103463, December 17, 2021)

The names of Chloe Williams and Jonathan D. Gilthorpe were inadvertently omitted from the author list. The author list and affiliations have now been corrected online. The authors apologize for this error.

