# Supplementary material for: Generation and analysis of innovative genomically humanized knockin SOD1, TARDBP (TDP-43), and FUS mouse models
Source: iScience. 2021 Nov 15;24(12):103463. doi: 10.1016/j.isci.2021.103463 (PMC8710557; doi:10.1016/j.isci.2021.103463)
Supplement: Document S1. Figures S1–S21 [file mmc1.pdf]

iScience, Volume *24*

Supplemental information

Generation and analysis of innovative genomically humanized knockin *SOD1*, *TARDBP* (TDP-43), and *FUS* mouse models

Anny Devoy, Georgia Price, Francesca De Giorgio, Rosie Bunton-Stasyshyn, David Thompson, Samanta Gasco, Alasdair Allan, Gemma F. Codner, Remya R. Nair, Charlotte Tibbit, Ross McLeod, Zeinab Ali, Judith Noda, Alessandro Marrero-Gagliardi, José M. Brito-Armas, Chloe Williams, Muhammet M. Öztürk, Michelle Simon, Edward O'Neill, Sam Bryce-Smith, Jackie Harrison, Gemma Atkins, Silvia Corrochano, Michelle Stewart, Jonathan D. Gilthorpe, Lydia Teboul, Abraham Acevedo-Arozena, Elizabeth M.C. Fisher, and Thomas J. Cunningham

Supplementary Figures

Xdrop indirect target locus enrichment and Nanopore sequencing to confirm allele integrity in humanised mice
Figure S1
Figure S2
Figure S3
Figure S4
Figure S5
Figure S6
Figure S7
Figure S8
Figure S9
Figure S10
Figure S11
Figure S12
Figure S13

Humanised mouse strains express human, not mouse, gene products
Figure S14
Figure S15
Figure S16
Figure S17
Figure S18
Figure S19

Homozygous hFUS mice display no motor or other overt phenotypes throughout aging, and maintain normal motor neuron counts in the lumbar spinal cord
Figure S20

(Bioinformatics commands for long-read sequencing alignments)
Figure S21

Figure S1. Brief overview of Xdrop locus capture; Related to STAR Methods. Using the *hFUS* locus as an example. High molecular weight DNA was encapsulated in Xdrop droplets; detection sequences (indicated in green) were designed to be spaced such that the size of the captured DNA would allow overlapping coverage; droplet PCR and staining followed by FACS isolated droplets from the target detection loci; droplet multiple displacement amplification to amplify target DNA; followed by DNA purification and Oxford Nanopore sequencing.

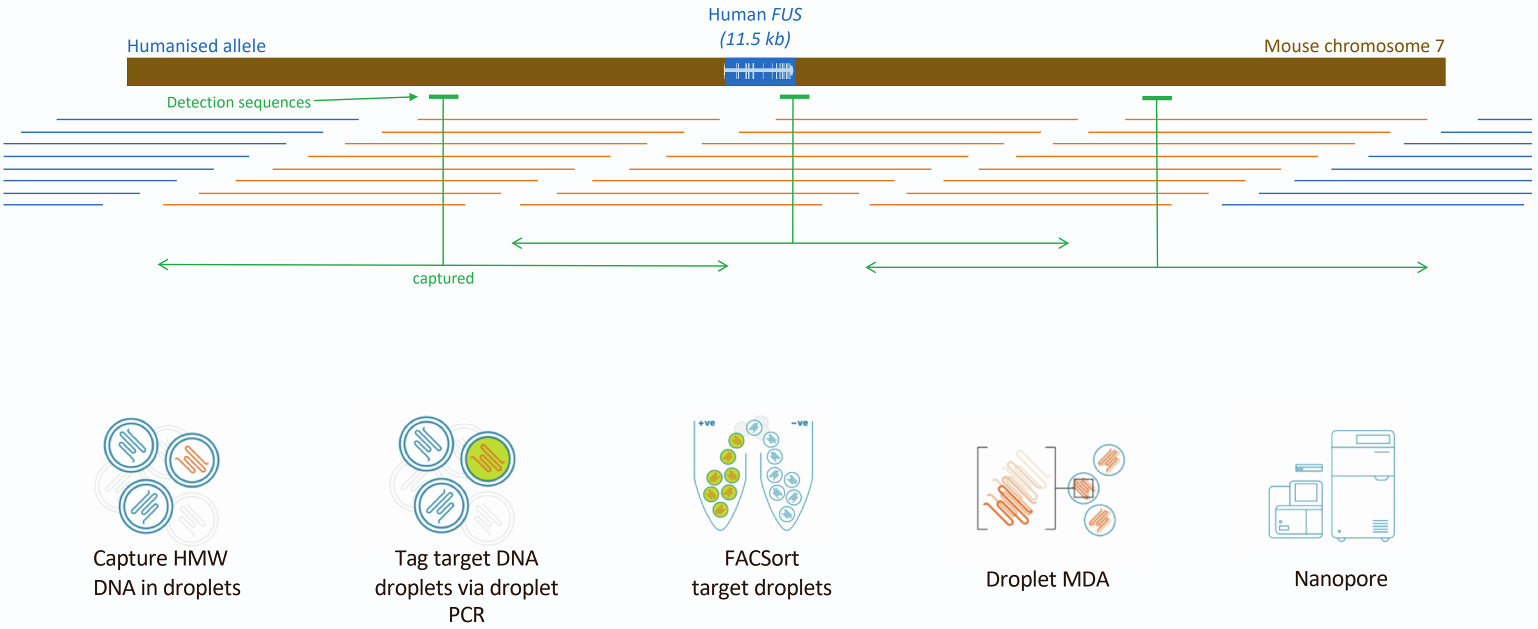

Figure S2. Alignment of *hSOD1/hSOD1* sequencing reads to 1 Mb *Sod1* C57BL/6J locus; Related to Figure 1. IGV visualization of minimap2 alignment to the GRCm38 C57BL/6J reference genome, zoomed in to 1 Mb surrounding the *Sod1* locus. Red asterisks note the positions of detection sequences, which broadly correspond to the peaks in coverage.

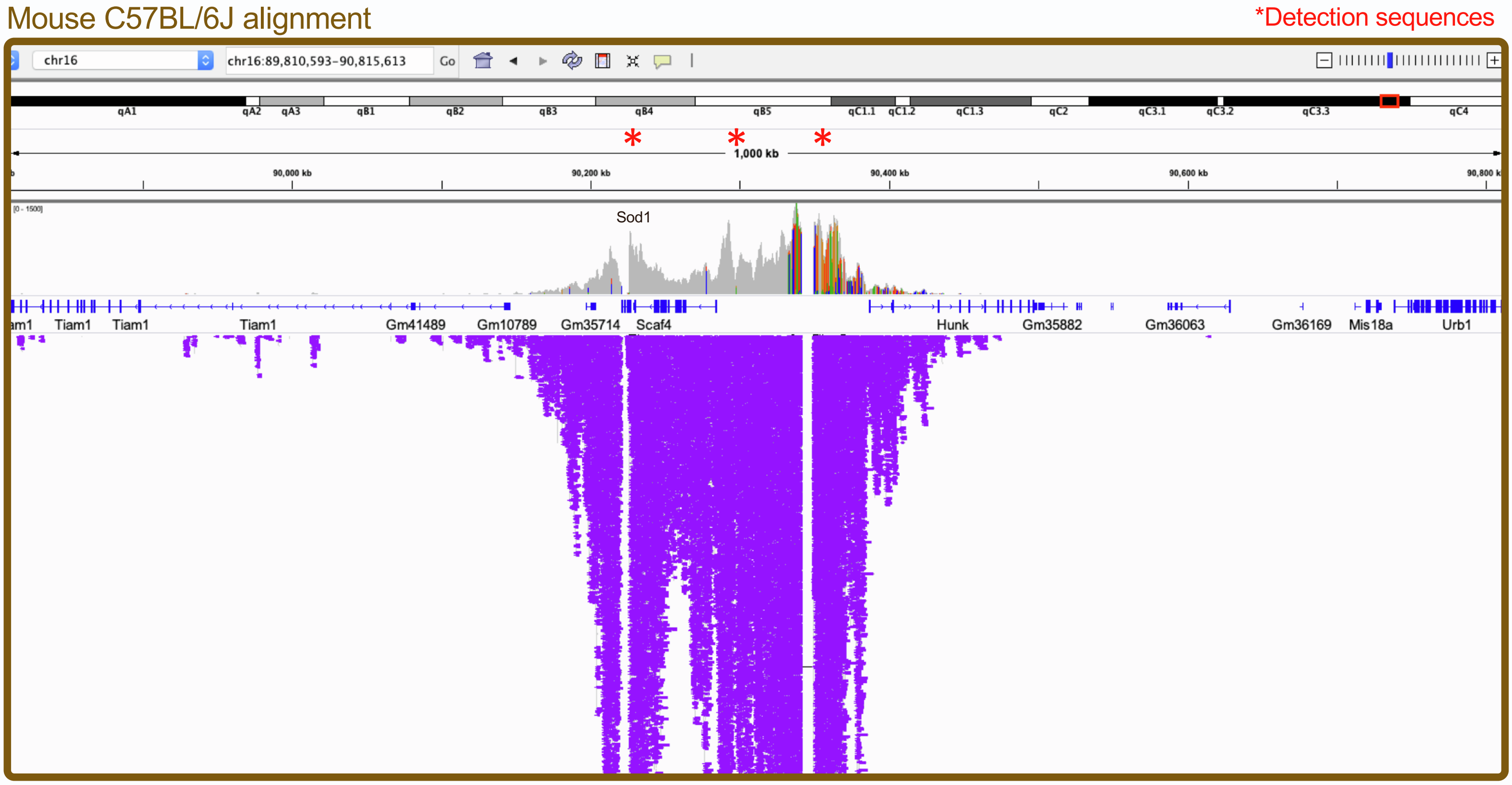

Figure S3. Alignment of *hTARDBP/hTARDBP* sequencing reads to 1 Mb *Tardbp* C57BL/6J locus; Related to Figure 2. IGV visualization of minimap2 alignment to the GRCm38 C57BL/6J reference genome, zoomed in to 1 Mb surrounding the *Tardbp* locus. Red asterisks note the positions of detection sequences, which broadly correspond to the peaks in coverage.

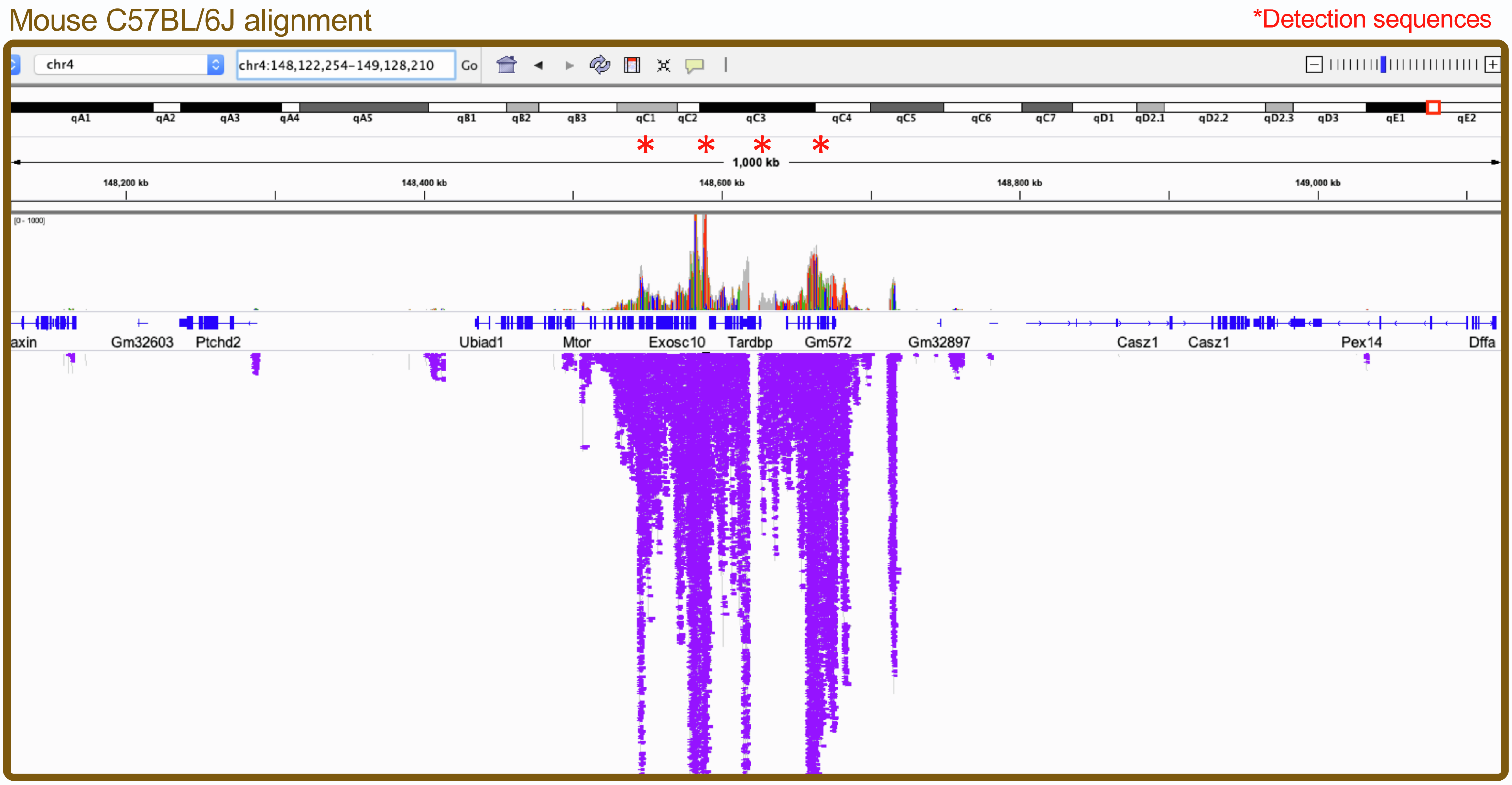

Figure S4. Alignment of *hFUS/hFUS* sequencing reads to 1 Mb *Fus* C57BL/6J locus; Related to Figure 3. IGV visualization of minimap2 alignment to the GRCm38 C57BL/6J reference genome, zoomed in to 1 Mb surrounding the *Fus* locus. Red asterisks note the positions of detection sequences, which broadly correspond to the peaks in coverage.

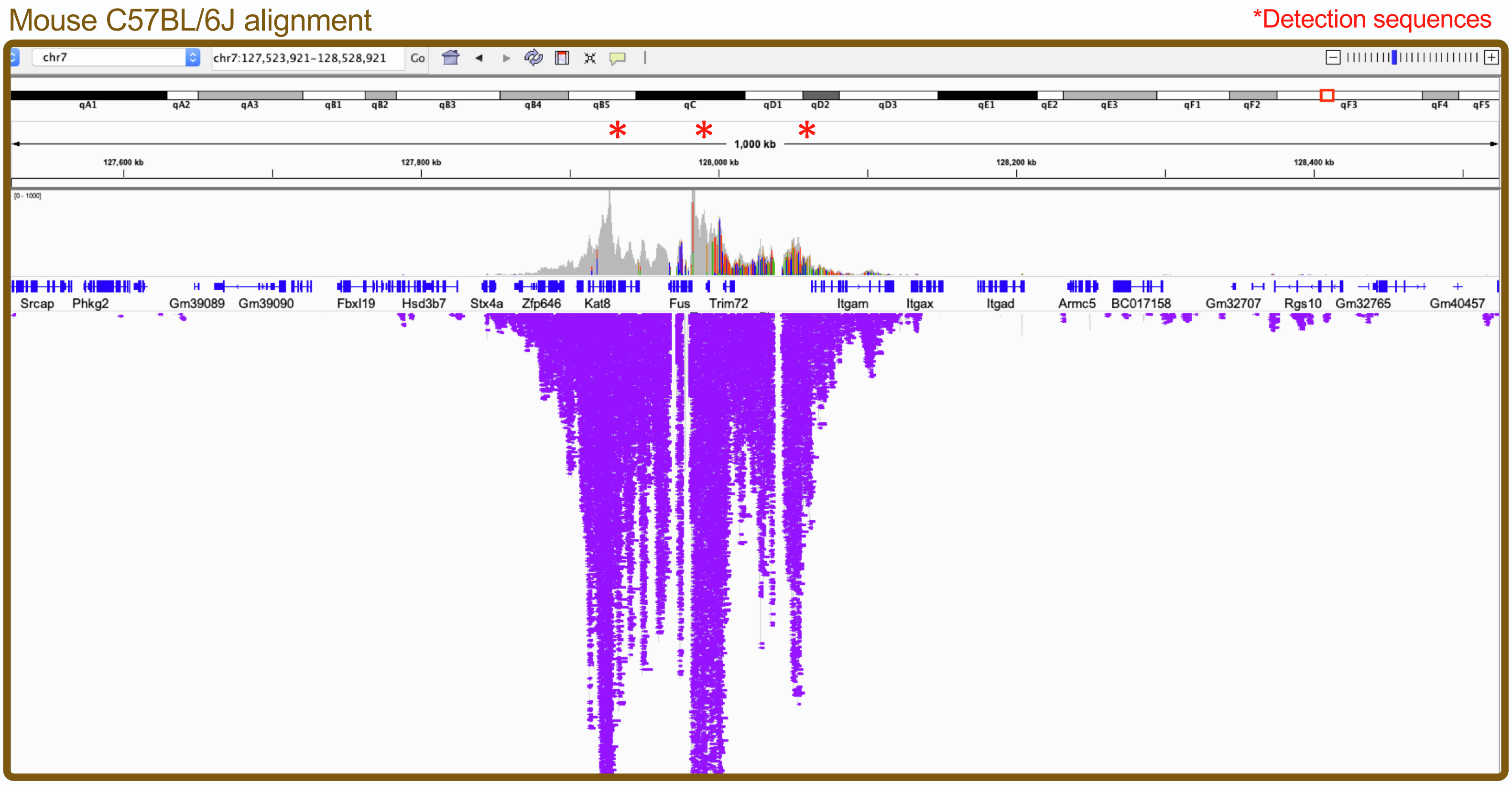

Figure S5. ***hSOD1* allele theoretical map alignment; Related to Figure 1.** Alignment (minimap2) of *hSOD1/hSOD1* mouse sequencing reads to a file of the intended humanised allele sequence (green outline top IGV panel); a single imperfect alignment feature (*h) maps the correct base in the majority, but is within a homopolymeric sequence and shows a higher error rate within reads. Engineered duplications (red and brown arrows) and engineered *loxP* and *FRT* site insertions are only evident as anomalies when aligning to human and mouse reference genomes (lower IGV panels). In the alignment to the human genome, a SNP is evident in intron 1 (#) that was known to be present in the targeting construct and is a known human polymorphism.

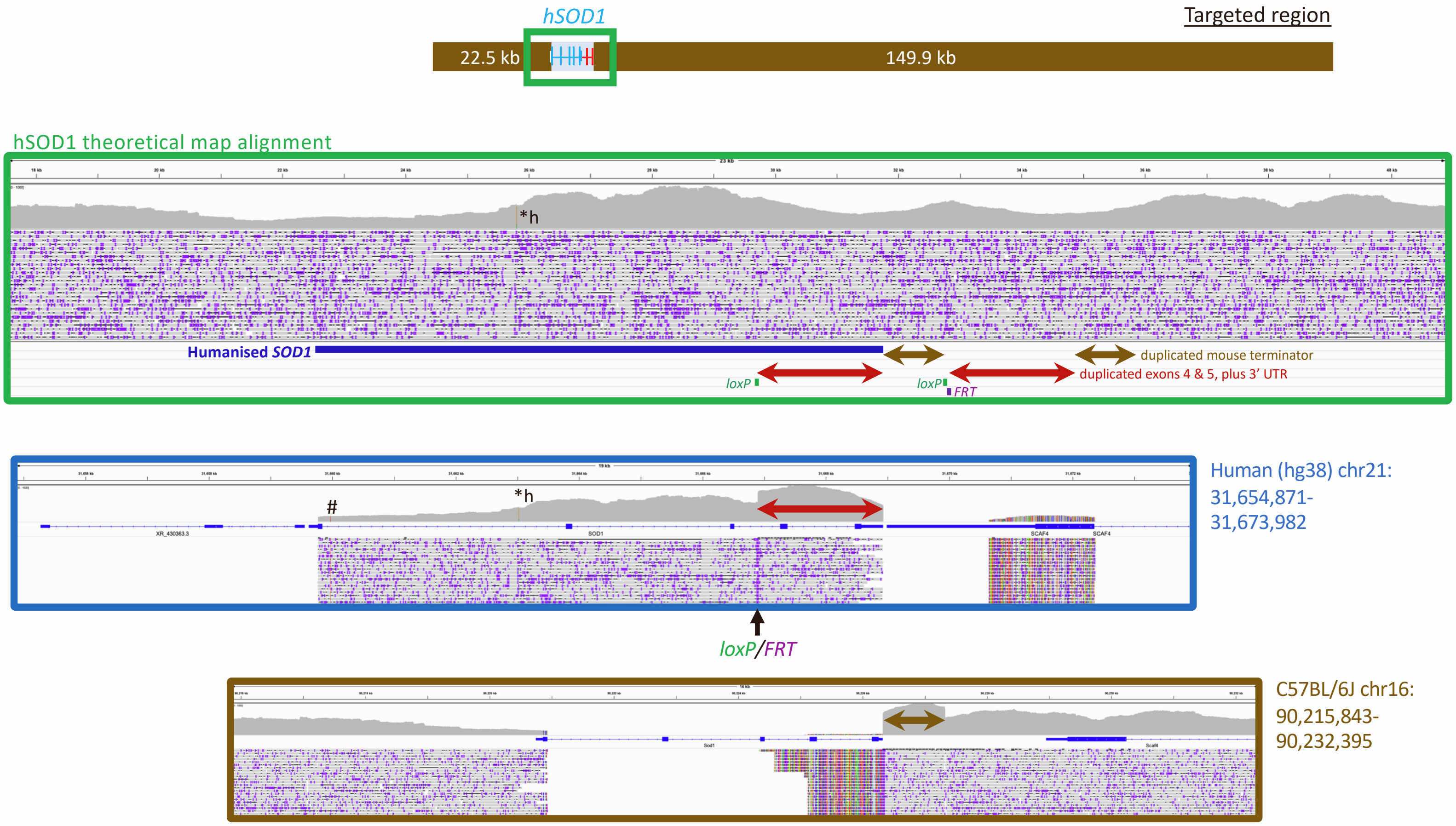

# = Known human SNP variant in the targeting construct that differs from the human reference genome
*h = imperfect alignment feature (homopolymeric sequence)

Figure S6. ***hTARDBP* allele theoretical map alignment; Related to Figure 2.** Alignment (minimap2) of *hTARDBP/hTARDBP* mouse sequencing reads to a file of the intended humanised allele sequence, including the frt-flanked selection cassette, which was not excised before sequencing (green outline top IGV panel). The selection cassette region is only evident as an anomaly when aligning to human reference genome (*sc; human reference genome alignments, bottom two blue- (minimap2) and red- (NGMLR) IGV panels). Numerous SNPs in the human *TARDBP* gene (#) are known human polymorphisms present in the targeting construct that differ from the human reference genome, and are only evident as anomalies when aligning to the human reference genome. Two more complex anomalies are denoted by red arrows in intron 2 and intron 5 (region X and Y), which show a number of base positions with high error rates (coloured lines, although the majority of reads map correctly). The intron 5 anomaly additionally shows aberrant sharp changes in coverage. Alignment with an alternative alignment software program, NGMLR (bottom IGV panel, red outline), resolves these latter anomalous features. The only remaining anomalies to account for map to homopolymeric sequences (*h).

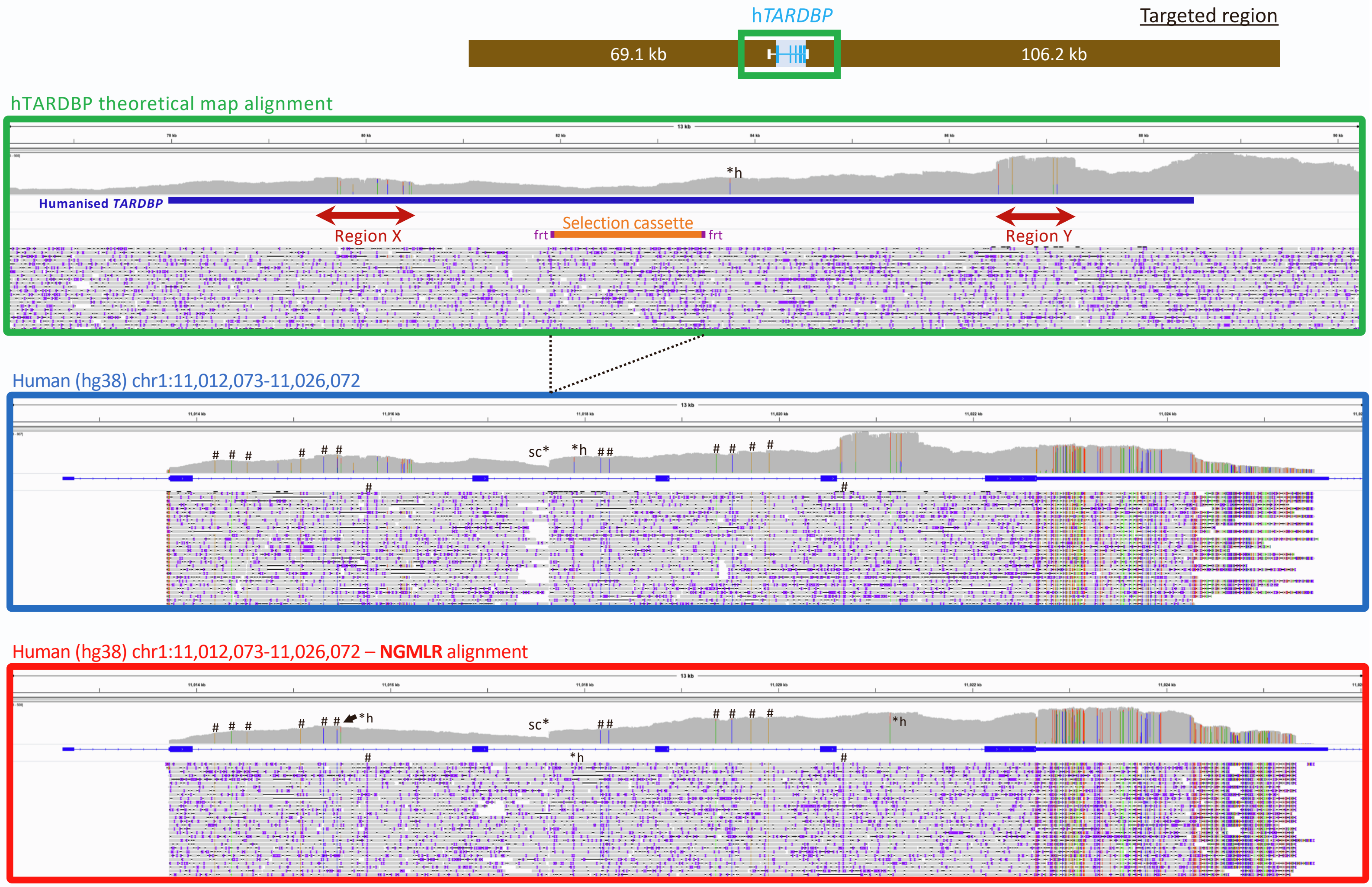

# = known human polymorphisms present in the targeting construct that differ from the human reference genome
*h = homopolymeric sequence
sc* = selection cassette

Figure S7. *hFUS* allele theoretical map alignment; Related to Figure 3. Alignment (minimap2) of *hFUS/hFUS* mouse sequencing reads to a file of the intended humanised allele sequence (green outline top IGV panel). A single imperfect alignment feature (asterisk) maps the correct base in the majority, but is within a repeating element region and shows a higher error rate within reads. Lower four panels show that engineered *loxP* and *FRT* site insertions are only evident as anomalies when aligning to human and mouse reference genomes; bottom and middle right IGV panels are theoretical alignment zooms as indicated; bottom and middle left show the equivalent zoomed regions in human and mouse alignments.

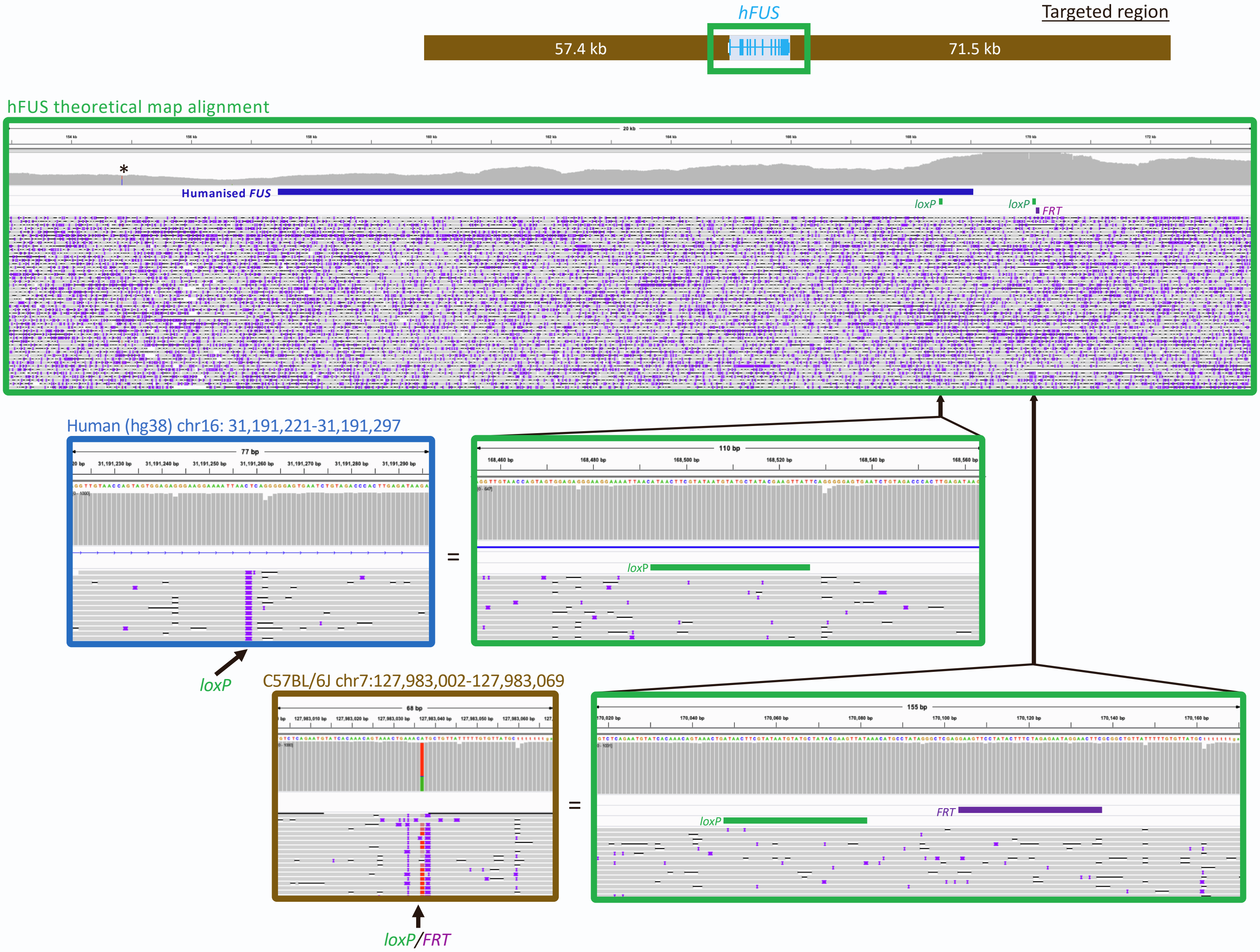

* = imperfect alignment feature (repeating element region)

Figure S8. Regions X and Y in *hTARDBP* introns 2 and 5 precisely map to the boundaries of SINE elements; Related to Figure 2. Screenshots from UCSC genome browser zoomed in on human introns 2 and 5, with SINE elements indicated by black bars, and regions X and Y indicated by arrows.

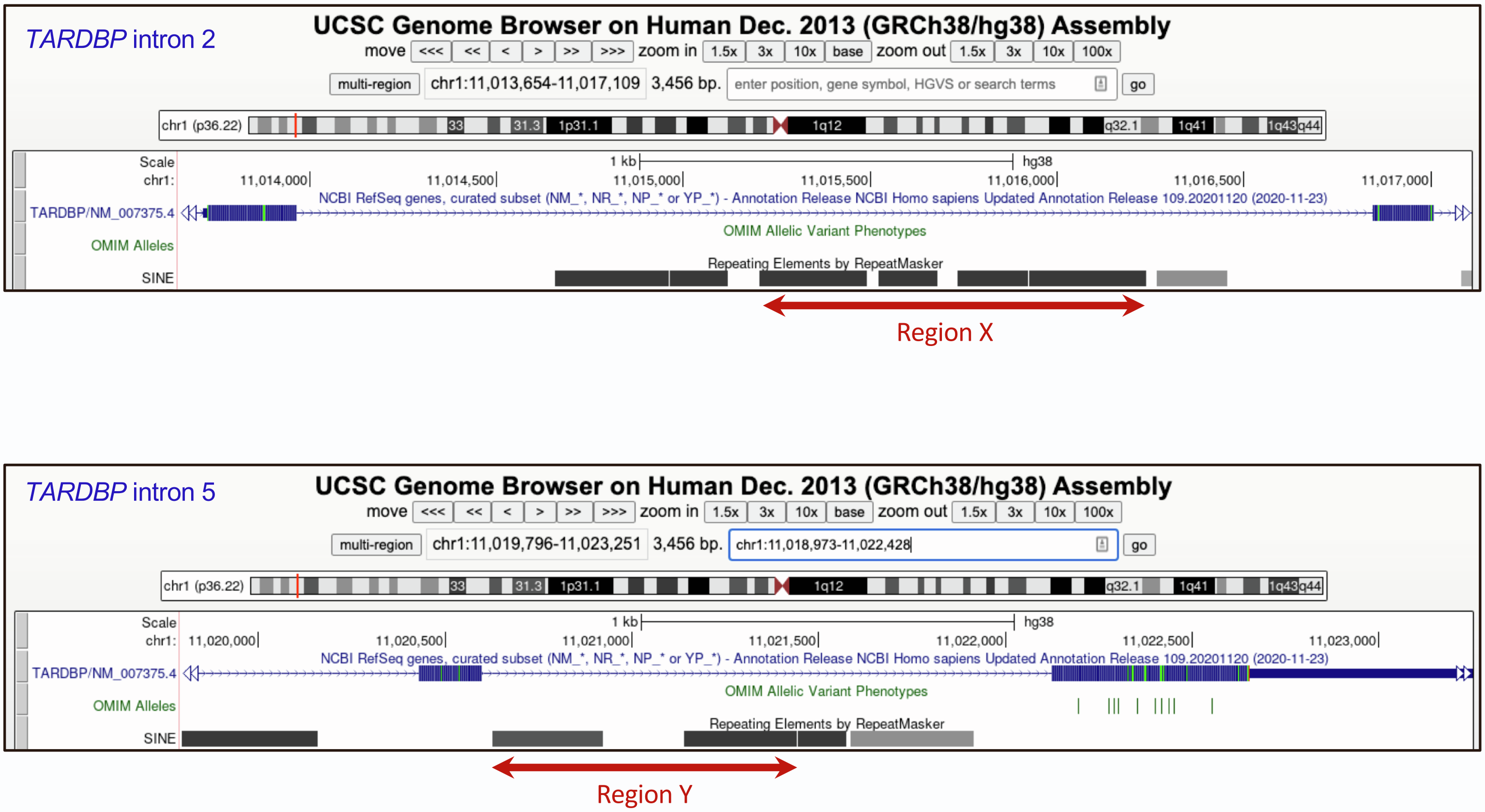

Figure S9. *hTARDBP* allele: B6 to 129 transition, 5'; Related to Figure 2. Comparison of B6 and 129 strain alignments of *hTARDBP/hTARDBP* sequencing reads to reveal the homologous recombination breakpoint in the 5' homology arm correspondent region, 11 kb upstream of *hTARDBP*. The dotted line marks the equivalent breakpoint position in the two alignments. Some imperfect (but not misaligned) alignment features are also highlighted, with examples given (**). Unannotated regions in the 129 reference genome are also highlighted.

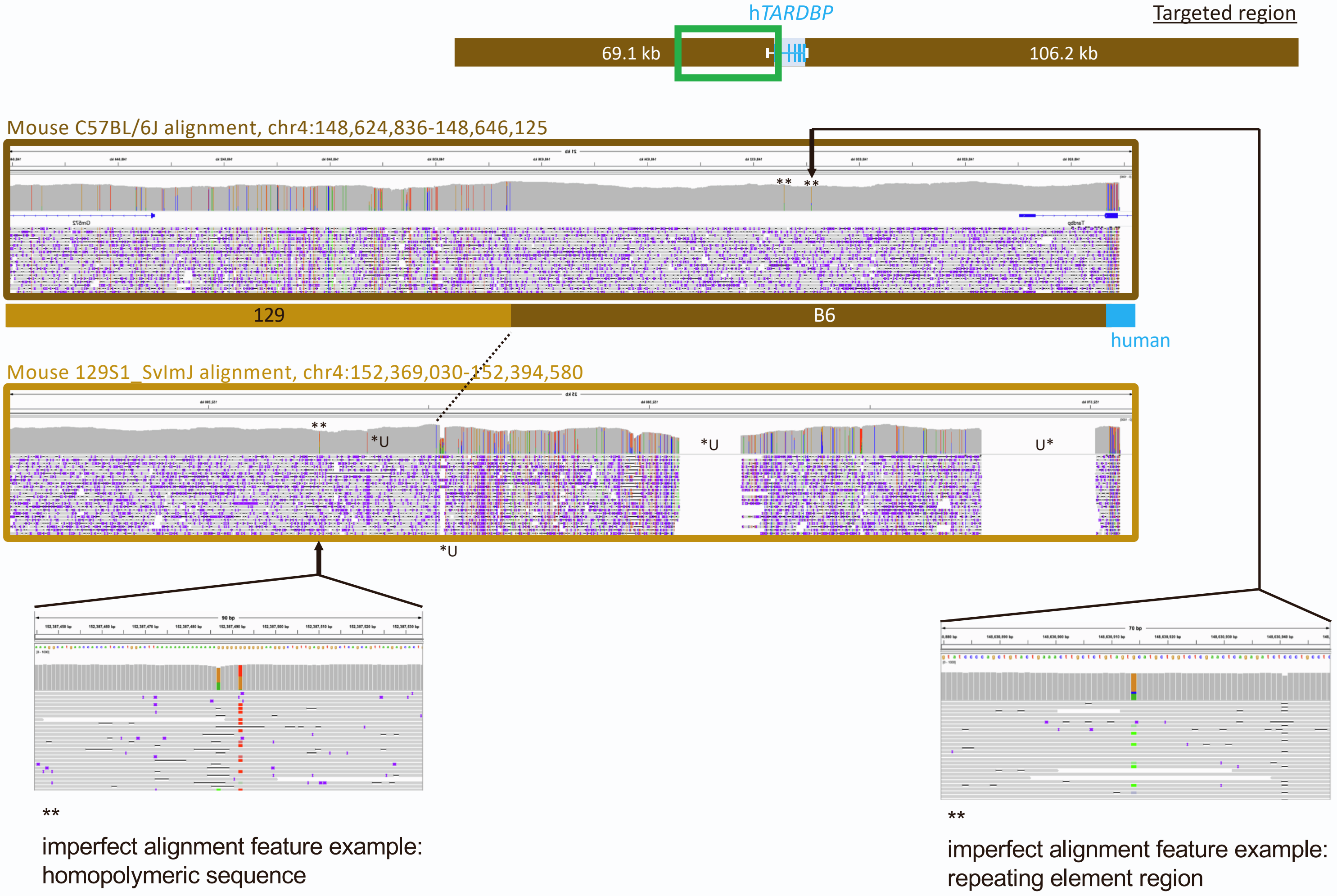

*U = unannotated region in 129 reference genome

** = imperfect alignment feature

Figure S10. *hTARDBP* allele: B6 to 129 transition, 3'; Related to Figure 2. Comparison of B6 and 129 strain alignments of *hTARDBP/hTARDBP* sequencing reads to reveal the homologous recombination breakpoint in the 3' homology arm correspondent region, 5.5 kb downstream of *hTARDBP*. The dotted line marks the equivalent breakpoint position in the two alignments. Lower panels highlight the breakpoint region at higher resolution. An unannotated region in the 129 reference genome is also highlighted.

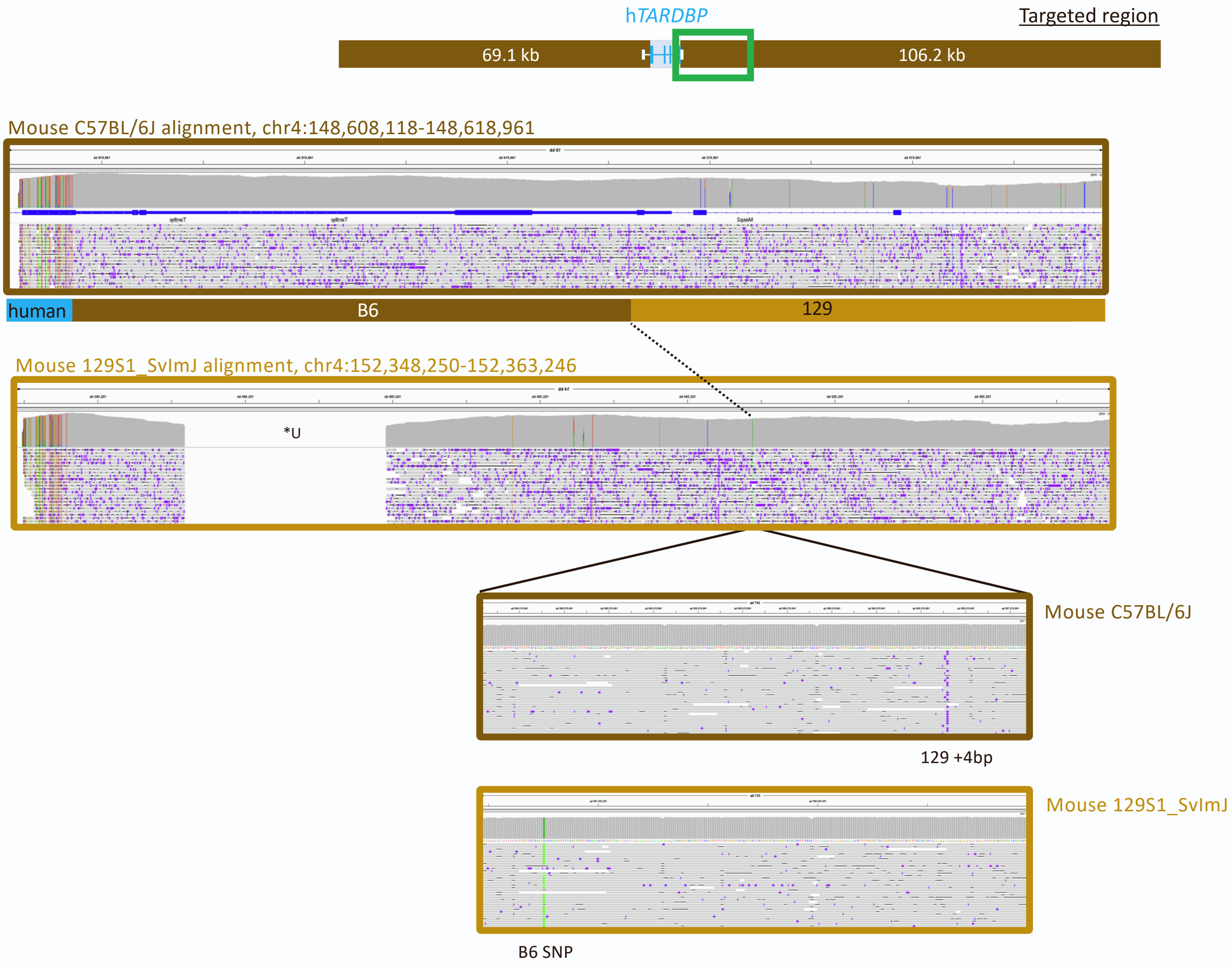

*U = unannotated region in 129 reference genome

Figure S11. *hFUS* allele: B6 to 129 transition, 5'; Related to Figure 3. Comparison of B6 and 129 strain alignments of *hFUS/hFUS* sequencing reads shows that the proximal ~30 kb of the 5' homology arm clearly maps precisely to the B6 reference genome and is derived from the targeting construct. The remaining distal portion of the 5' homology arm (only the 3' extremity of this region is shown, marked by a B6-specific insertion, indicated by arrow) has no appreciable divergence between strains to make a clear determination. Some imperfect (but not misaligned) alignment features are also highlighted, with an example given (**). Unannotated regions in the 129 reference genome are also highlighted.

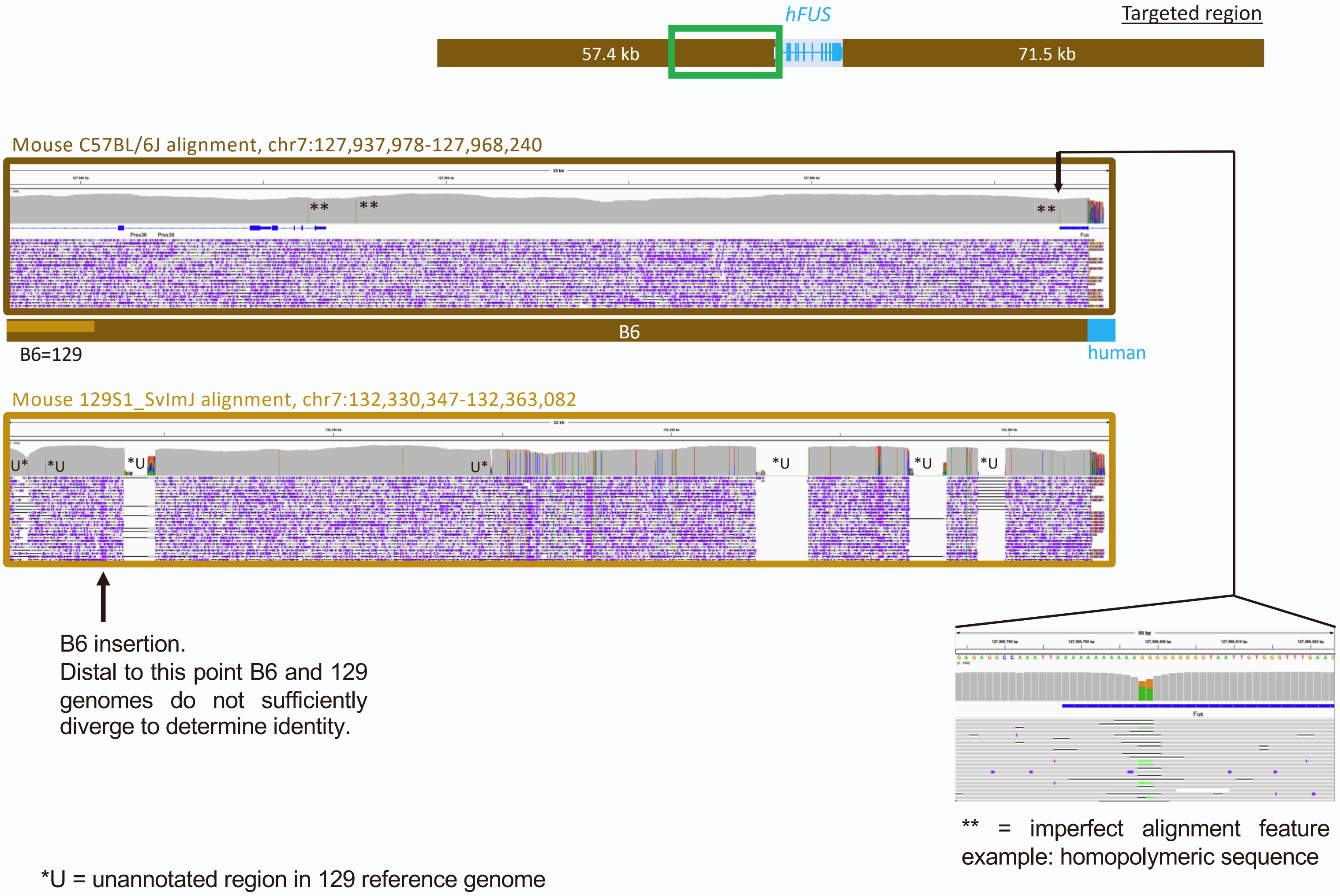

Figure S12. *hFUS* allele: B6 to 129 transition, 3'; Related to Figure 3. Comparison of B6 and 129 strain alignments of *hFUS/hFUS* sequencing reads shows the 3' homologous recombination breakpoint can be narrowed to a region 10-15 kb downstream of *hFUS*. The proximal ~15 kb of the 3' homology arm equivalent region clearly maps to the B6J reference genome, with the exception of a single SNP that maps to strain 129 (*129) ~10 kb downstream of *hFUS*. The remaining distal portion of the 3' homology arm correspondent region region maps to strain 129. Imperfect alignment features are also highlighted.

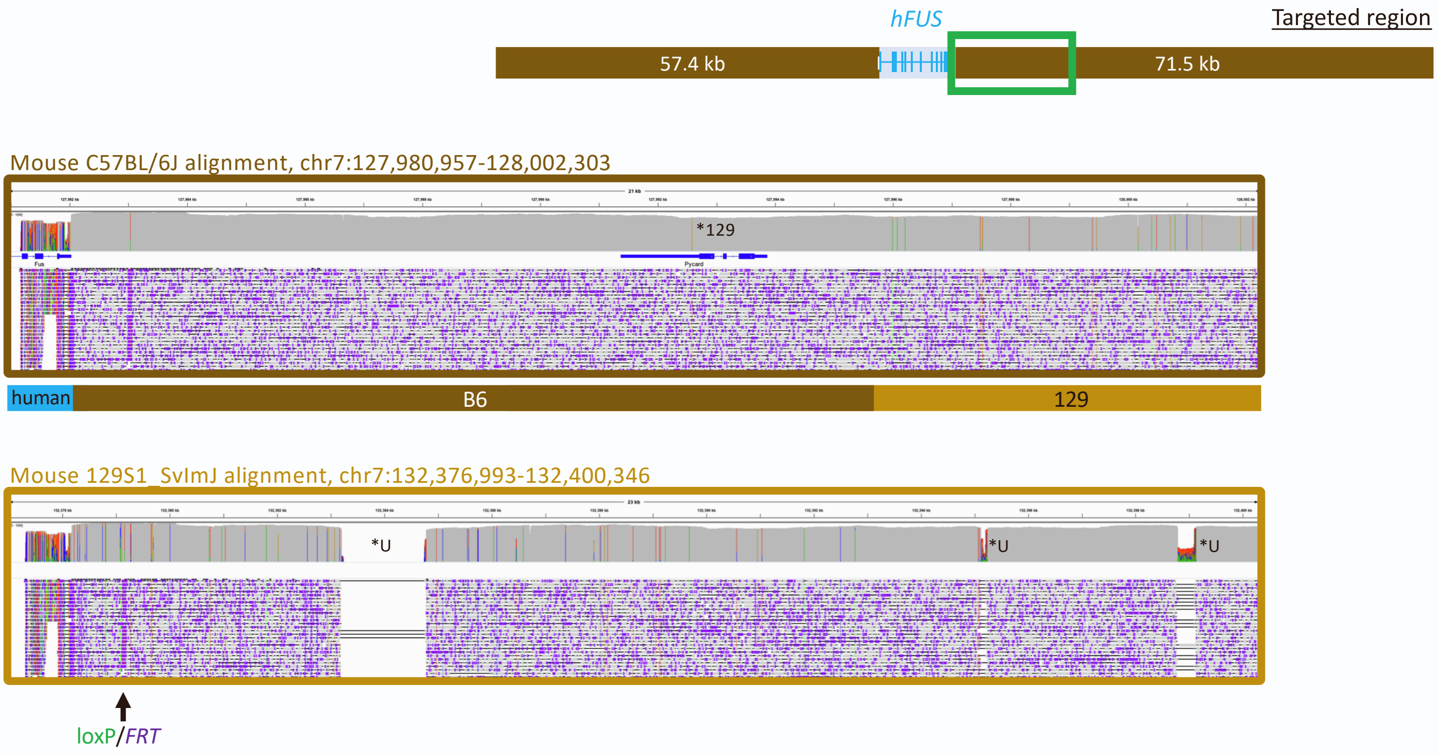

*U = unannotated region in 129 reference genome
*129 = 129-specific SNP within a region of B6 identity

Figure S13. *hSOD1* allele: B6 to 129 transition, 3'; Related to Figure 1. Comparison of B6 and 129 strain alignments of *hSOD1/hSOD1* sequencing reads shows the 3' homologous recombination breakpoint can be narrowed to a region ~100 kb downstream of *hSOD1*. The proximal ~100 kb of the 3' homology arm correspondent region (only the 3' extremity of this region is shown, marked by a B6-specific insertion, indicated by arrow) clearly maps to the B6 reference genome, whilst the remaining distal portion of the 3' homology arm correspondent region maps to strain 129. Unannotated regions in the 129 reference genome are also highlighted.

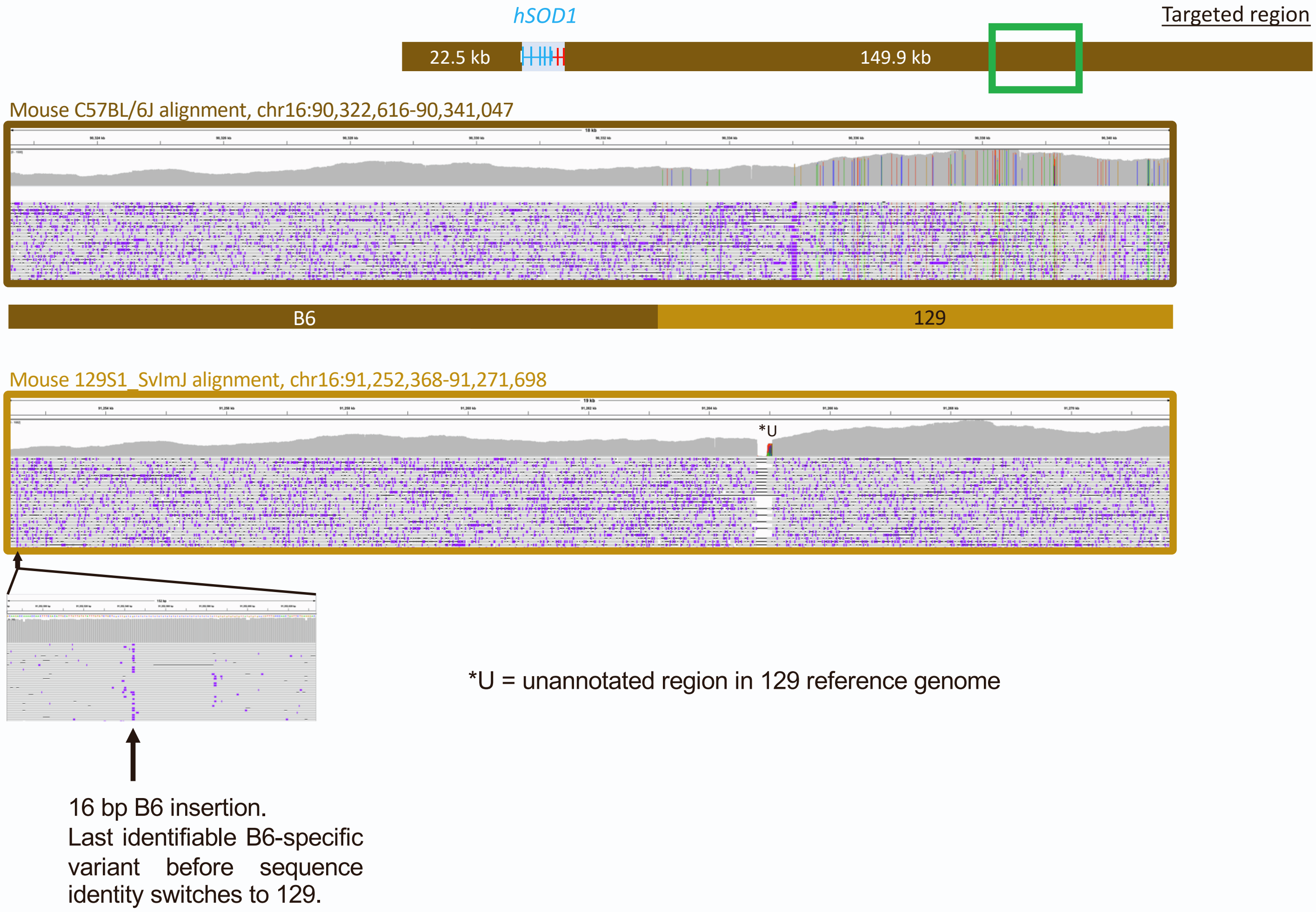

Figure S14. Immunofluorescent staining of SOD1 in lumbar spinal cord; Related to Figure 4. Transverse sections are from 6-month old male mice using a pan-mouse-human SOD1 antibody. Scale bar 20μm.

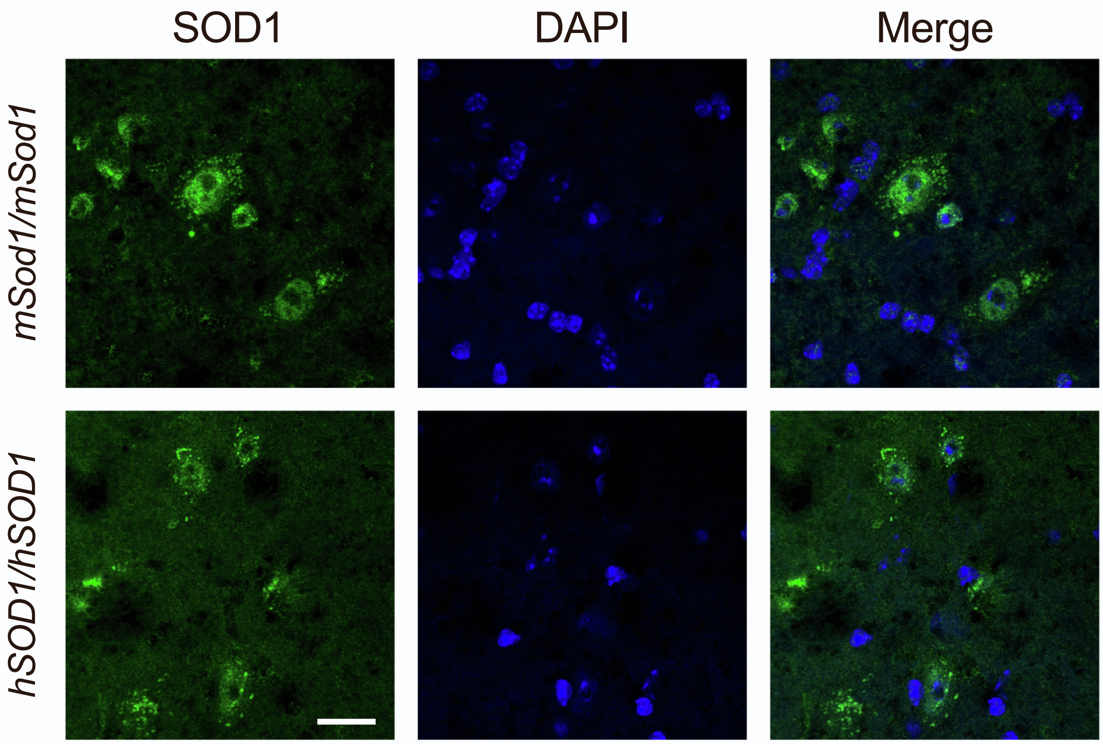

Figure S15. Splicing of TDP-43 target exons and TDP-43 nuclear localization are unaffected in h*TARDBP* mice; Related to Figure 5. (A) RT-PCR analysis of *Sort1* exon 18 and *Eif4h* exon 5 inclusion from brain and spinal cord of 14-week old females (n=3 per genotype). Lower bands = exon excluded, upper bands = exon included. No statistically significant differences ($p>0.05$) were found for any comparison. Mean ± SD, One-way ANOVA with Dunnett's post hoc test. **(B)** Immunofluorescent staining of TDP-43 in cortex from 10-week old male mice using a pan-mouse-human TDP-43 antibody. TDP-43 staining is predominantly nuclear. Scale bar = 10 µm.

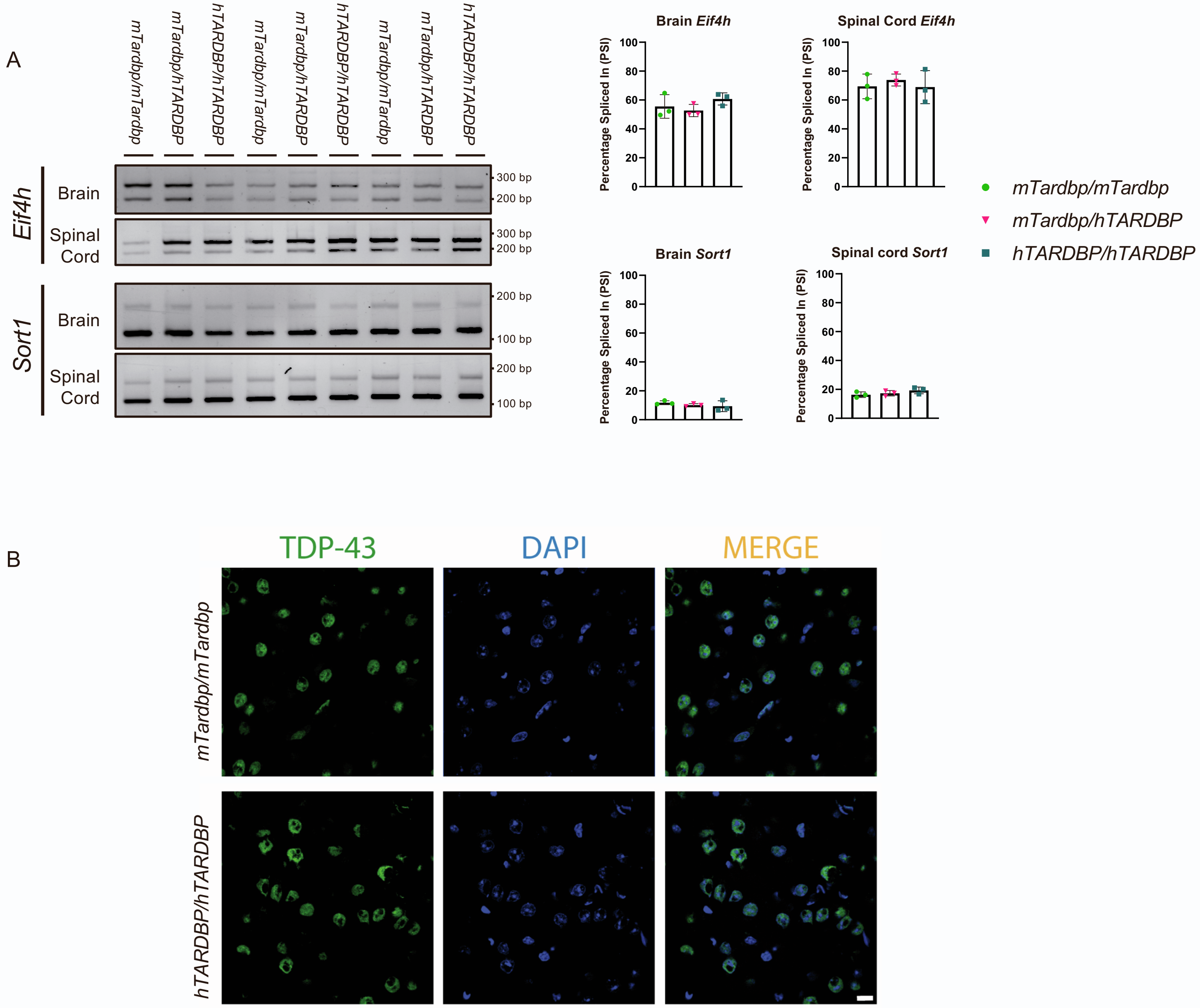

Figure S16. *hFUS* mRNA and protein expression in 3-month old male tissues and immunofluorescence at 18 months; Related to Figure 6. Expression of *FUS* mRNA and protein in 3 month old male hFUS mice. (A) qRT-PCR using conserved mouse-human *Fus/FUS* primers was carried out in spinal cord and tibialis anterior (TA) muscle (n=4 per genotype). Data presented as mean ± SD, ns=not statistically significant, *=p≤0.05 calculated using One-way ANOVA with Dunnett's post hoc test. (B) Immunobloting using pan-mouse-human FUS antibody in male spinal cord and tibialis anterior (TA) muscle tissue (n=4 per genotype per sex). Includes gels used for quantification of FUS protein expression. Data presented as mean ± SD, ns=not statistically significant, calculated using One-way ANOVA with Dunnett's post hoc test. (C) qRT-PCR using conserved mouse-human *Fus/FUS* primers to determine levels of intron 6 and intron 7 containing FUS transcripts, relative to correctly spliced *FUS* (D) Immunofluorescent staining of FUS in lumbar spinal cord of 18-month old mice using a pan-mouse-human FUS antibody. FUS staining is predominantly nuclear with diffuse staining in both genotypes. Scale bar = 50 µm.

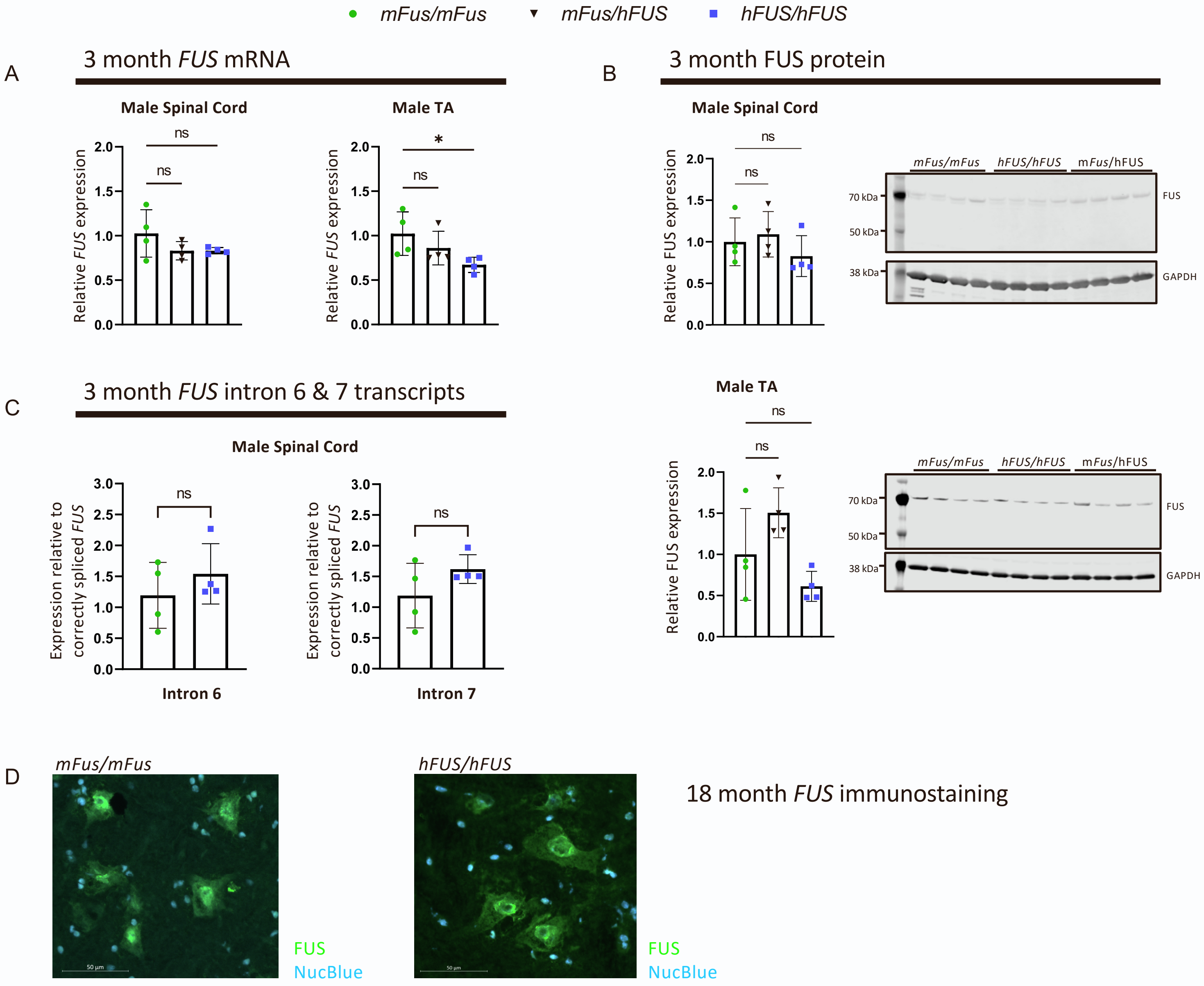

Figure S17. hFUS protein expression analysed longitudinally in spinal cord tissue; Related to Figure 6. Immunoblot using pan-mouse-human FUS antibody in male spinal cord tissue from mice aged 3 months (n=3 per genotype) and 18 months (n=4 per genotype). Includes gel used for quantification of FUS protein expression using total protein stain for normalisation. Data presented as mean ± SD, ns = not statistically significant, **=p≤0.01, calculated using 2way ANOVA with Šídák's multiple comparisons post hoc test.

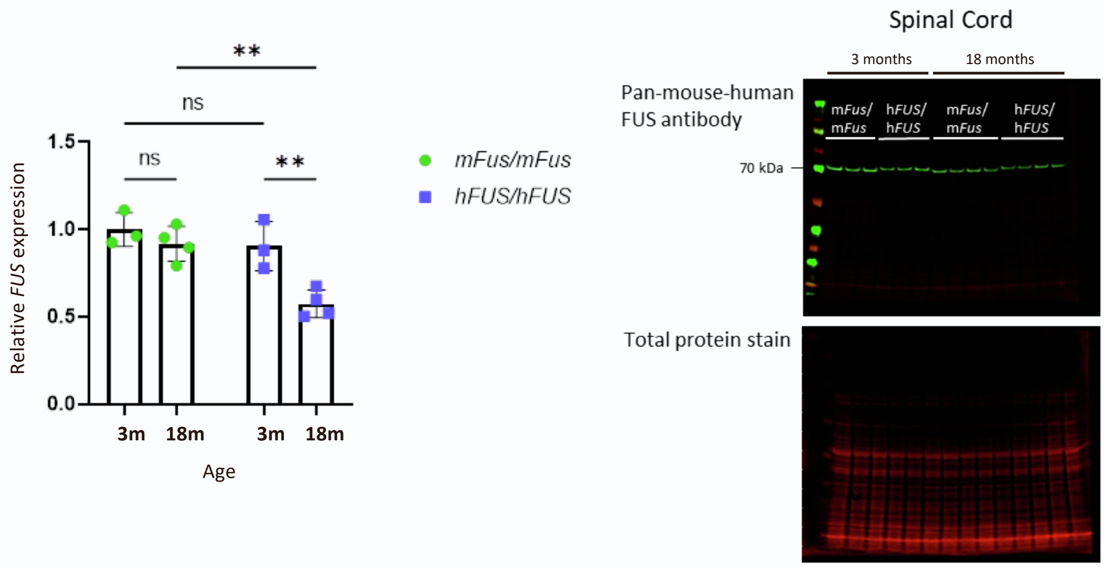

Figure S18. Images of immunoblots used for FUS expression quantification in Figure 6, and for SOD1 expression quantification in figure 4. Red asterisk indicates data point excluded due to anomaly on gel.

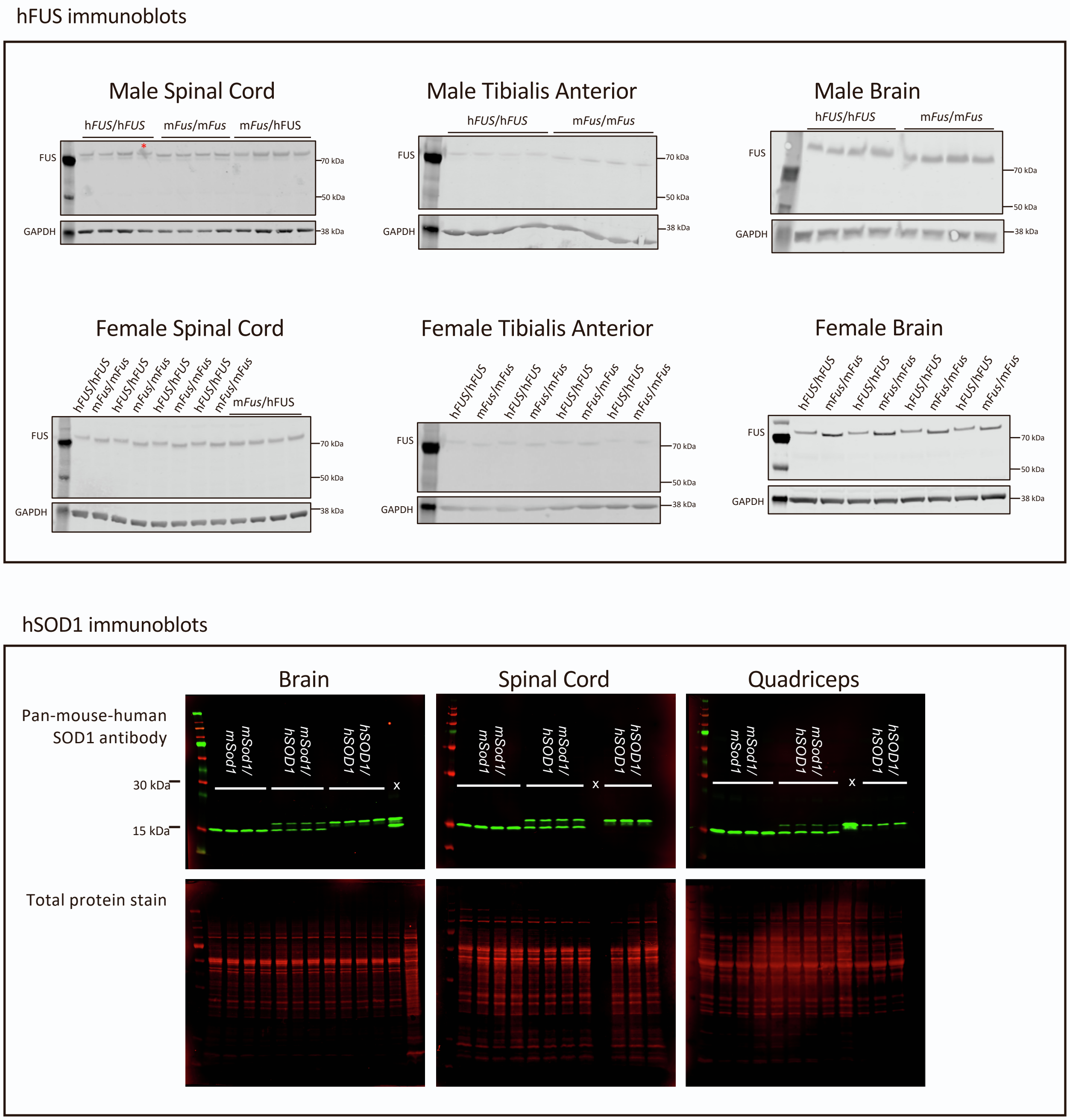

Figure S19. *hSOD1* and *hFUS* muscle morphology; Related to Figure 4. (A, B) Hematoxylin and eosin staining of tibialis anterior muscle from 4-month old *hSOD1* mice and 18-month old *hFUS* mice (n=2-3 per genotype). Scale bar = 100μm. (C, D) Quantification of fiber size and central nucleation in tibialis anterior muscle from *hSOD1* and *hFUS* mice. Mean ± SD, student's t-test.

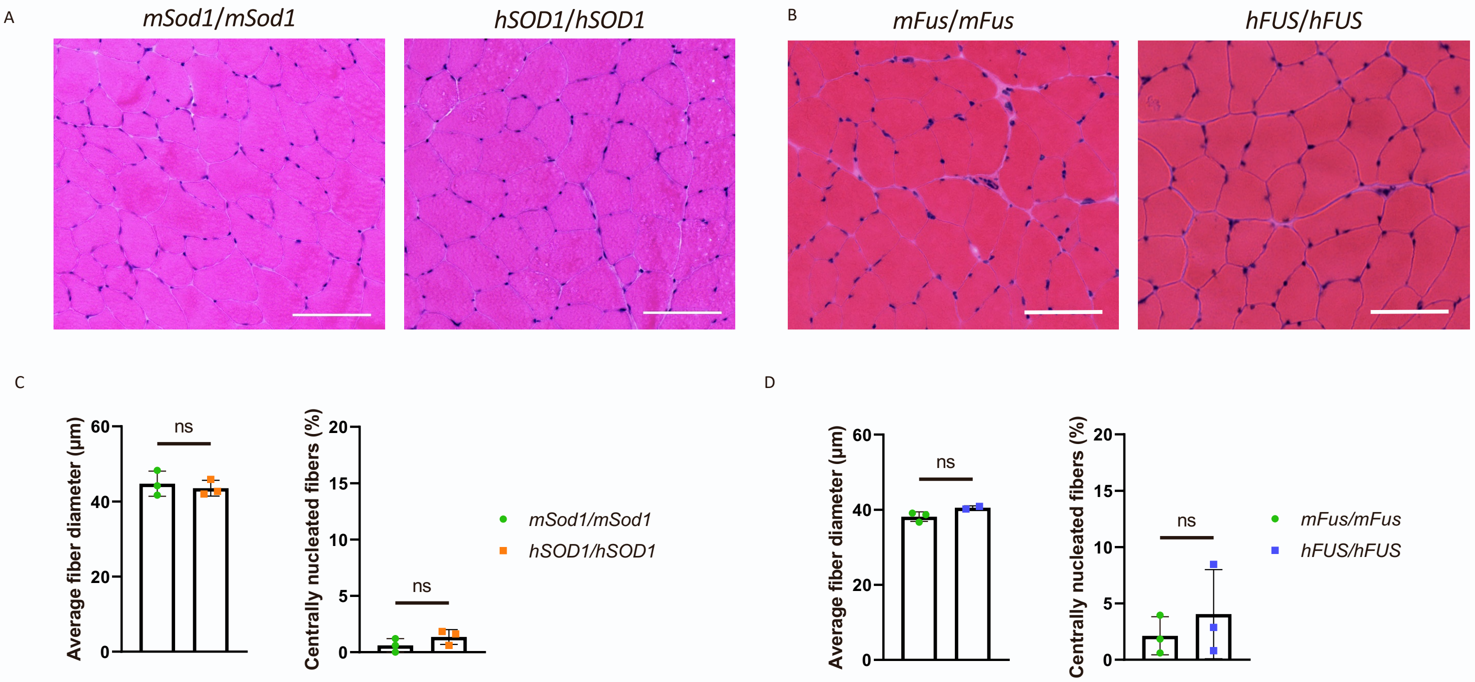

Figure S20. Modified SHIRPA analyses in *hFUS* mice; Related to Figure 7. Modified SHIRPA analyses at 3 and 18 months in *mFus/mFus* and *hFUS/hFUS* mice (3 months: n=17,15,16,13; male *mFus/mFus,* male *hFUS/hFUS,* female *mFus/mFus,* female *hFUS/hFUS,* 18 months: n=12,10,14,11; male *mFus/mFus,* male *hFUS/hFUS,* female *mFus/mFus,* female *hFUS/hFUS*). No significant differences were found for any parameter calculated using Fisher's exact test or chi square tests.

	3 months		18 months	
	mFus/mFus *(n = 33)*	***hFUS/hFUS*** *(n = 28)*	***mFus/mFus*** *(n = 26)*	***hFUS/hFUS*** *(n = 21)*
Normal activity	33	26	25	17
Increased/decreased activity	0	2	1	4
Feet on bars	33	27	26	19
Feet falling through bars	0	1	0	2
Normal gait	29	21	20	17
Abnormal gait	4	7	4	3
Gait not studied	0	0	2	1
Limb grasp absent	33	28	17	16
Limb grasp present	0	0	9	5
Contact righting present	33	28	24	19
Contact righting absent	0	0	2	2
Normal negative geotaxis	33	27	26	18
Slipping/loss of grip	0	1	0	3
Falls	0	0	0	0

Figure S21. Bioinformatics alignment commands; Related to Figures 1,2,3.

Alignment using minimap2:

```
minimap2 -ax map-ont ReferenceGenome.fa SequencingFiles.fastq.gz > AlignedReads.sam
```

Alignment using NGMLR:

```
ngmlr -t 4 -r ReferenceGenome.fa -q SequencingFiles.fastq.gz -o AlignedReads.sam -x ont
```

Conversion, indexing, and sorting:

```
samtools view -bS -q 50 AlignedReads.sam > AlignedReads.bam
samtools sort AlignedReads.bam -o AlignedReads.sorted.bam
samtools index AlignedReads.sorted.bam
```
