# Supplementary material for: Generation and analysis of innovative genomically humanized knockin SOD1, TARDBP (TDP-43), and FUS mouse models
Source: iScience. 2021 Nov 15;24(12):103463. doi: 10.1016/j.isci.2021.103463 (PMC8710557; doi:10.1016/j.isci.2021.103463)
Supplement: Table S1. Primer and probe sequences [file mmc2.docx]

**Table S1. Primer and Probe sequences.**

| **Assay** | **Primer/probe** | **Sequence** |
| --- | --- | --- |
| Xdrop; dPCR enrichment; | forward primer | GAATGTCATCCCCTGGTGCA |
| hSOD1 | reverse primer | GTGCCCTTTACTTGGTGCAG |
| Xdrop; qPCR enrichment validation; | forward primer | TGATCCCAGGTGTTTGAGCT |
| hSOD1 | reverse primer | GCCTGTGTTTGGGGTGTAGA |
| Xdrop; dPCR enrichment; | forward primer | GCCCATGGCTTCCTTTACAC |
| Sod1 3’ homology arm proximal | reverse primer | AAGCAAAAGTGGCAGGAAGC |
| Xdrop; qPCR enrichment validation; | forward primer | TATGTCTCCAGGCCAGGCT |
| Sod1 3’ homology arm proximal | reverse primer | GCCTTGTCACGCCTTGTAAC |
| Xdrop; dPCR enrichment; | forward primer | GCCCAACAACTCGGTAGGAA |
| Sod1 3' homology arm distal | reverse primer | CGTAGCCTCAGTCAGCAAGT |
| Xdrop; qPCR enrichment validation; | forward primer | AGTATGGGTTGCAGGCTGTC |
| Sod1 3' homology arm distal | reverse primer | CGCTACAGTGGGATGGAATGT |
| Xdrop; dPCR enrichment; | forward primer | TAGAGCCCAGAGTCAGCCTT |
| Fus 5' homology arm | reverse primer | TACCTGCAGCTGATTGGCAA |
| Xdrop; qPCR enrichment validation; | forward primer | GGTCCTGGAAGCATGGTCAA |
| Fus 5' homology arm | reverse primer | CCCGTACTTTTCCTGCCACT |
| Xdrop; dPCR enrichment; | forward primer | CTAGGTGGATGGTGGCGAAT |
| FUS 3' UTR | reverse primer | ACAAATCAGGCCAACATGGTG |
| Xdrop; qPCR enrichment validation; | forward primer | CGGGGAGAAGGCCAAATGAT |
| FUS 3' UTR | reverse primer | TGCTGTGCAAGACAATTTCTGG |
| Xdrop; dPCR enrichment; | forward primer | CTCCCAGCTGAGCTTTGAGA |
| Fus 3' homology arm | reverse primer | AGTTGTTGGAGGCTTGGTGA |
| Xdrop; qPCR enrichment validation; | forward primer | CGGTTTGGTTTTCACGGTGT |
| Fus 3' homology arm | reverse primer | ATGAGCCAAGCAGAGGACAG |
| Xdrop; dPCR enrichment; | forward primer | CCCACGTGGTTTCTGTAGCA |
| Tardbp 5' homology arm distal | reverse primer | CCATCCAGACCCGTAACCTG |
| Xdrop; qPCR enrichment validation; | forward primer | GGAGGTGAGTGGATCCTGGA |
| Tardbp 5' homology arm distal | reverse primer | CACCTAGGGAGCTGATGCAC |
| Xdrop; dPCR enrichment; | forward primer | TTGGGTCTCCCTGGACAGAG |
| Tardbp 5' homology arm proximal | reverse primer | TCACAGAGCAAACAAATCCCA |
| Xdrop; qPCR enrichment validation; | forward primer | GCATGCTTATGCCTAAGTGACA |
| Tardbp 5' homology arm proximal | reverse primer | TCTGGCAGCTACCACATTCC |
| Xdrop; dPCR enrichment; | forward primer | TGGTGTGACTGCAAACTTCCT |
| Human TARDBP gene | reverse primer | AGGGCCAAAGACTTCAACAAG |
| Xdrop; qPCR enrichment validation; | forward primer | TGTGTGAGTATGTGCACTTTTAGA |
| Human TARDBP gene | reverse primer | CGAACAAAGCCAAACCCCTTT |
| Xdrop; dPCR enrichment; | forward primer | TCCAAGGACTGATGCTGGAC |
| Tardbp 3' homology arm | reverse primer | AAAGGGGCCATGGAAGTGAC |
| Xdrop; qPCR enrichment validation; | forward primer | CCCACCCGACATCTTGAACA |
| Tardbp 3' homology arm | reverse primer | AGTGTCAACAGACCCAGGGA |

**Table S1. Primer and Probe sequences (continued).**

| Mouse Sod1 copy number qPCR assay | forward primer | GTACCAGTGCAGGACCTCAT |
| --- | --- | --- |
|  | reverse primer | AGCGTGCTGCTCACCTCT |
|  | 5’-FAM probe | AACATGGTGGCCCGGCGGATG |
| Human SOD1 copy number qPCR assay | forward primer | GTGCAGGTCCTCACTTTAATCC |
|  | reverse primer | CCAGAAAGCTATCGCCATTATTACAAG |
|  | 5’-FAM probe | CCAAAGGATGAAGAGAGGTAACAAGATGC |
| Mouse Fus copy number qPCR assay | forward primer | GGCGGTTGTGTGTGTATGTG |
|  | reverse primer | AACATGGACCCATTCTTCAGAAAG |
|  | 5’-FAM probe | CATCATTTTAGTTAAATTCTGTTTCC |
| Human FUS copy number qPCR assay | forward primer | CCCAGCAGGAACTGGAATACAG |
|  | reverse primer | AACATGGACCCATTCTTCAGAAAG |
|  | 5’-FAM probe | TTCTGTCATGGGGAAATTCTGTTTCCC |
| RT-PCR human SOD1 specific assay | forward primer | TCGTCTTGCTCTCTCTGGTC |
|  | reverse primer | CAGGCCTTCAGTCAGTCCTT |
| mouse Sod1 specific RT-PCR assay | forward primer | AACCAGTTGTGTTGTCAGGAC |
|  | reverse primer | CCACCATGTTTCTTAGAGTGAGG |
| human TARDBP specific RT-PCR assay | forward primer | ATGACTGAGGATGAGCTGCG |
|  | reverse primer | CACAAAGAGACTGCGCAATCTG |
| mouse Tardbp specific RT-PCR assay | forward primer | CCATTCAGAGCTTTTGCCTTC |
|  | reverse primer | AGCTCCACCCCCTCTACTG |
| human FUS specific RT-PCR assay | forward primer | CGGTCGTCTGGAACTTTGTT |
|  | reverse primer | CCATAACCGCCACCACTG |
| mouse Fus specific RT-PCR assay | forward primer | CGGTCGTCTGGAACTTTGTT |
|  | reverse primer | CATAACCACCACCGCCAC |
| Pan human-mouse SOD1 qRT-PCR assay | forward primer | CACAGGCTATGGAACTCAGTCA |
|  | reverse primer | CCACCTTTGCCCAAGTCATC |
| Pan human-mouse TARDBP qRT-PCR assay | forward primer | GAGCCTTTGAGAAGCAGAAA |
|  | reverse primer | CCACCTGGATTACCACCAAA |
| Pan human-mouse FUS qRT-PCR assay | forward primer | CACAGGCTATGGAACTCAGTCA |
|  | reverse primer | GCCATAGCCAGGGTAGGAG |
| Mouse Masp2 qRT-PCR assay | forward primer | ACCGCTGCGAGTATGACTTT |
|  | reverse primer | CCTGTGAACGGCTTCTCATT |
| Mouse s16 qRT-PCR assay | forward primer | TTCTGGGCAAGGAGCGATT |
|  | reverse primer | GATGGACTGTCGGATGGCA |
| Mouse Gapdh qRT-PCR assay | forward primer | CGGCCGCATCTTCTTGTG |
|  | reverse primer | CCGACCTTCACCATTTTGTCTAC |
| Mouse Sortilin RT-PCR splicing assay | forward primer | CAGGAGACAAATGCCAAGGT |
|  | reverse primer | TGGCCAGGATAATAGGGACA |
| Mouse Eif4h RT-PCR splicing assay | forward primer | TGGATTCAGGAAAGGTGGAC |
|  | reverse primer | GGTCTCTGTGCTCGTTCCTC |
| Pan human-mouse FUS qRT-PCR splicing assay | forward primer | GGTGGCTTCAATAAATTTGGTGG |
| (ex7-10, introns spliced out) | reverse primer | AGTTTCCCTGTCTGTGTACAAA |
| Pan human-mouse FUS qRT-PCR splicing assay | forward primer | TGTGTGCGTGTGTTTAATGC |
| (intron 6 retention) | reverse primer | GTCACCTTTCATACCTGTGGC |
| Pan human-mouse FUS qRT-PCR splicing assay | forward primer | GGTGGCTTCAATAAATTTGGTGG |
| (intron 7 retention) | reverse primer | ACACGCTAAGACATCTGCAA |
